# PDHA1 Hyperactivation‐Mediated Glucose Metabolism Reprogramming Fuels Neutrophil NETosis and Promotes Acute Lung Injury

**DOI:** 10.1002/advs.78307

**Published:** 2026-10-14

**Authors:** Yu‐Biao Liu, Wen‐Jing Zhong, Chen‐Yu Zhang, Meng‐Rui Chen, Nan‐Shi‐Yu Yang, Han‐Xi Sha, Yan‐Ling Qiu, Hui Chen, Jin‐Tong Yang, Li‐Ying Liang, Jian‐Bing Xiong, Shao‐Kun Liu, Cha‐Xiang Guan, Jia‐Xi Duan, Yong Zhou

**Affiliations:** ^1^ Department of Geriatric Respiratory and Critical Care Medicine Xiangya Hospital Central South University Changsha Hunan China; ^2^ School of Nursing Shandong Xiehe University Jinan Shandong China; ^3^ Xiangya Nursing School Central South University Changsha Hunan China; ^4^ Department of Physiology Xiangya School of Basic Medical Sciences Central South University Changsha Hunan China; ^5^ Basic and Clinical Research in Major Respiratory Disease Key Laboratory of General University of Hunan Province Changsha Hunan China; ^6^ National Experimental Teaching Demonstration Center For Medical Function Changsha Hunan China; ^7^ Department of Emergency Xiangya Hospital Central South University Changsha Hunan China; ^8^ Department of Pulmonary and Critical Care Medicine The Second Xiangya Hospital Central South University Changsha Hunan China; ^9^ School of Basic Medicine Ningxia Medical University Yinchuan Ningxia China; ^10^ National Clinical Research Center for Geriatric Disorders Xiangya Hospital Central South University Changsha Hunan China

**Keywords:** acute lung injury, NETosis, neutrophils, PDHA1, selective metabolic reprogramming

## Abstract

Neutrophil extracellular trap (NET) formation, termed NETosis, is a key driver of lung tissue injury in acute lung injury/acute respiratory distress syndrome (ALI/ARDS). However, the precise mechanisms that trigger neutrophil NETosis remain incompletely understood. Here, we observed that neutrophils from both ALI mice and ARDS patients exhibited NETosis alongside a pronounced selective metabolic shift, characterized by enhanced oxidative phosphorylation (OXPHOS) and attenuated glycolytic activity. Phosphoproteomic profiling revealed that this shift was driven by aberrant activation of pyruvate dehydrogenase E1 subunit alpha (PDHA1), resulting from its dephosphorylation at Ser293. Inhibiting PDHA1 activity, using either the inhibitor 3‐fluoropyruvate (3‐FP) or genetic knockdown, effectively reversed this selective metabolic reprogramming and suppressed NETosis. We further constructed neutrophil‐targeted 3‐FP‐loaded albumin nanoparticles (3‐FP‐BSA NPs). These nanoparticles were specifically taken up by activated neutrophils, markedly inhibited NETosis, and alleviated lipopolysaccharide‐induced ALI in mice. Mechanistically, RNA‐seq revealed the enrichment of the AMPK in neutrophils during NETosis. AMPK phosphorylated PDHA1 at Ser314, which disrupted the PDHA1‐pyruvate dehydrogenase kinase 1 (PDK1) interaction and inhibited PDHA1 phosphorylation at Ser293. Ultimately, PDHA1 hyperactivation‐driven selective metabolic reprogramming precipitated mitochondrial stress and mtROS accumulation, thereby initiating NETosis. This study defines a targetable metabolic pathway in neutrophils and advances a specific PDHA1‐nanotherapeutic strategy against ALI/ARDS.

## Graphical abstract

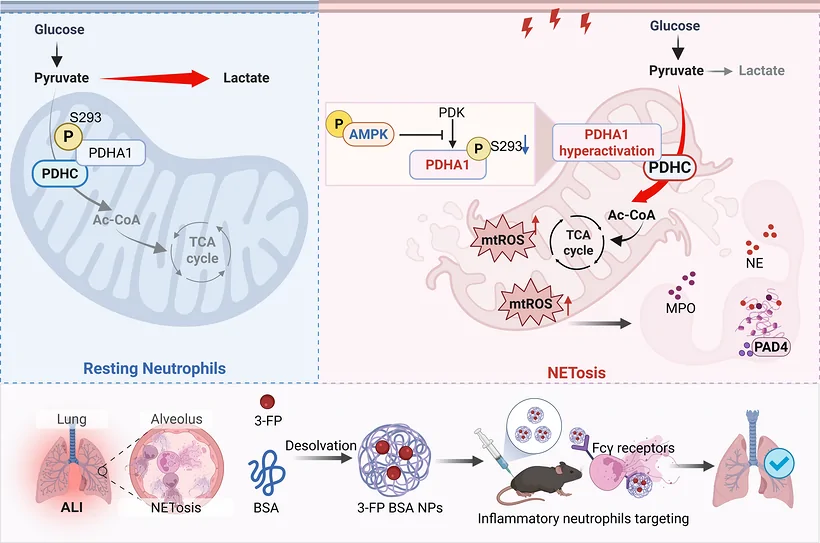

**Abbreviations**
ALI: acute lung injury
ARDS: acute respiratory distress syndrome
ATRA: all‐trans retinoic acid
ATP5A: ATP synthase F1 subunit alpha
AMPK: adenosine monophosphate‐activated protein kinase
APACHE: Acute Physiology and Chronic Health Evaluation
BCS: bovine serum
BCA: bicinchoninic acid
BMI: body mass index
BSA: bovine serum albumin
citH3: citrullinated histone H3
CRP: C‐reactive protein
CXCR2: C‐X‐C motif chemokine receptor 2
CSF3R: colony stimulating factor 3 receptor
Co‑IP: co‑immunoprecipitation
dsDNA: double‐stranded DNA
DAMP: damage‐associated molecular pattern
DAD: diffuse alveolar damage
DMSO: dimethyl sulfoxide
DCA: dichloroacetate
ELISA: Enzyme‐linked immunosorbent assay
FPKM: fragments per kilobase of transcript per million mapped reads
FCGR3B: Fc gamma receptor IIIb
GSEA: Gene set enrichment analysis
HTSeq: high‐throughput sequence analysis in Python
HCs: healthy controls
HRP: horseradish peroxidase
HIF‐1α: hypoxia‐inducible factor 1α
IF: immunofluorescence
IPATH: interactive Pathways Explore
KEGG: Kyoto Encyclopedia of Genes and Genomes
LDH: lactate dehydrogenase
LPS: lipopolysaccharide
Ly6G: lymphocyte antigen 6 complex
MPO: myeloperoxidase
MRM: multiple reaction monitoring mode
mtROS: mitochondrial ROS
MTCO1: cytochrome c oxidase subunit 1
Micro‐CT: Micro‐computed tomography
NPs: nanoparticles
NADH: nicotinamide adenine dinucleotide
NDUFB8: NADH dehydrogenase beta subcomplex subunit 8
NETs: neutrophil extracellular traps
NE: neutrophil elastase
NLRP3: NLR family pyrin domain‐containing 3
NINJ1: ninjurin‐1
NADPH: nicotinamide adenine dinucleotide phosphate
OXPHOS: oxidative phosphorylation
OCR: the oxygen consumption rate
PAD4: peptidyl arginine deiminase 4
PDK1: pyruvate dehydrogenase kinase 1
PDHC: pyruvate dehydrogenase complex
PDHA1: pyruvate dehydrogenase E1 component subunit alpha
PBS: phosphate‐buffered saline
PCT: procalcitonin
PMA: phorbol 12‐myristate 13‐acetate
PPI: protein‑protein interaction
PVDF: polyvinylidene fluoride
PCA: Principal component analysis
ROS: reactive oxygen species
Real‐time PCR: Real‐time quantitative polymerase chain reaction
SDS: sodium dodecyl sulfate
SDHB: succinate dehydrogenase subunit B
SOFA: Sequential Organ Failure Assessment
TCA: tricarboxylic acid cycle
TE: Tris‐ethylenediaminetetraacetic acid
TNF‐α: tumor necrosis factor‐alpha
UQCRC2: cytochrome b‐c1 complex subunit 2
2,6‐DCPIP: 2,6‐dichlorophenolindophenol
7‐AAD: 7‐Aminoactinomycin D.

## 1 Introduction

Acute lung injury (ALI) and its severe form, acute respiratory distress syndrome (ARDS), represent life‐threatening conditions characterized by dyspnea, profound hypoxemia, decreased lung compliance, and diffuse bilateral infiltrates on chest radiography [1, 2]. Although the 2012 Berlin definition reclassified the spectrum of lung injury by categorizing ALI as mild ARDS [3], the term ALI continues to be widely used in preclinical and translational research. Over the past decade, improvements in supportive care, particularly lung‐protective ventilation and conservative fluid management, have contributed to better patient outcomes. Nevertheless, mortality remains substantial, especially in moderate to severe cases, highlighting the persistent gap in effective pharmacotherapies [4]. The complex pathophysiology, involving dysregulated inflammation, neutrophil‐mediated injury, disruption of endothelial and epithelial barriers, and impaired alveolar fluid clearance, continues to drive investigative efforts toward identifying novel molecular targets and therapeutic strategies [4].

Neutrophils are the key amplifiers of the ARDS immune cascade [5]. Upon activation, neutrophils release neutrophil extracellular traps (NETs), a process known as NETosis [6]. When this immune response is not spatiotemporally contained, it can disrupt the alveolar‐capillary barrier, promote edema formation, and lead to loss of aerated tissue, severe hypoxemia, organ failure, and ultimately death [7]. The accumulation and clustering of neutrophils at sites of tissue injury represent classic hallmarks of acute inflammation [8]. NETosis causes tissue and vascular damage by releasing damage‐associated molecular patterns (DAMPs), such as citrullinated histone H3 (citH3), myeloperoxidase (MPO), neutrophil elastase (NE), and cell‑free double‑stranded DNA (dsDNA) [9]. Our previous study found that NETs act as DAMPs, inducing diffuse alveolar damage and compromising the air‐blood barrier [10]; Furthermore, inhibiting ninjurin‐1 (NINJ1) oligomerization suppresses NETs release and attenuates ALI [11]. Therefore, targeting the process and products of NETosis is critical for treating ALI/ARDS. Current research on neutrophil NETosis mechanisms primarily involves calcium sparks, oxidative stress, and the activation of various kinases [12]. However, the triggering mechanisms of neutrophil NETosis during acute lung injury remain far from fully elucidated.

Glucose metabolic reprogramming has emerged as a critical regulator of inflammatory responses. Classical glucose metabolic reprogramming is defined by enhanced aerobic glycolysis and suppressed oxidative phosphorylation (OXPHOS), initially identified in cancer cells as the Warburg effect [13]. Our previous studies revealed that macrophage glucose metabolism reprogramming drives inflammatory activation [14], a mechanism similarly observed in T cells, where increased aerobic glycolysis supports proliferation and survival [15]. Neutrophils exhibit a distinct metabolic profile among immune cells, characterized by high glycolytic flux and restricted mitochondrial respiration [16]. Under physiological conditions, they predominantly utilize aerobic glycolysis for ATP generation. However, in tumor microenvironments, neutrophils shift to altered anaerobic glycolysis, mitochondrial metabolism, and amino acid metabolism to perform their immunosuppressive functions and facilitate tumor progression [17]. Despite these observations, the changes in glycolytic enzymes and metabolites during neutrophil NETosis and their functional relevance remain poorly defined.

The pyruvate dehydrogenase complex (PDHC) serves as a key metabolic node that irreversibly converts pyruvate to acetyl‐CoA, thereby bridging cytosolic glycolysis with mitochondrial OXPHOS [18]. This multienzyme complex localizes to the mitochondrial matrix and comprises three core subunits: the rate‐limiting E1 component (pyruvate dehydrogenase, consisting of catalytic PDHA and regulatory PDHB subunits), E2 (dihydrolipoyl transacetylase, DLAT), and E3 (dihydrolipoyl dehydrogenase, DLD). PDHA1 contains the principal catalytic site and is finely controlled by reversible phosphorylation [19]. In particular, phosphorylation at Ser293 acts as a major inhibitory switch that attenuates PDHC activity [20]. Under hypoxic conditions, Ser293 phosphorylation represses PDHC function, favoring glycolytic flux and promoting oncogenic progression in lung cancer [21]. This post‐translational modification not only reduces enzymatic efficiency but also impairs downstream phosphorylation events that depend on PDHA1 activity. Such regulatory mechanisms contribute to chemotherapy resistance by enabling metabolic adaptation, underscoring PDHA1 as a potential target in cancer treatment [22]. Nevertheless, the role of PDHA1 in neutrophil NETosis remains poorly investigated, despite its well‐established regulatory role in cancer metabolism.

Our study demonstrated that neutrophils in both ALI mice and ARDS patients underwent a pronounced selective metabolic reprogramming. Phosphoproteomic profiling revealed that this shift was driven by aberrant activation of PDHA1 following its dephosphorylation at Ser293. We further established that PDHA1 hyperactivation enhanced OXPHOS, disrupted mitochondrial homeostasis, and elevated mitochondrial reactive oxygen species (mtROS) production, thereby promoting NETosis. Notably, neutrophil‐targeted albumin nanoparticles loaded with a PDHA1 inhibitor significantly mitigated lipopolysaccharide (LPS)‐induced ALI in mice. Mechanistically, the activation of adenosine monophosphate‐activated protein kinase (AMPK) induced the phosphorylation of PDHA1 at Ser314, which disengaged pyruvate dehydrogenase kinase 1 (PDK1) and permitted PDHA1 dephosphorylation at Ser293, ultimately facilitating NETosis. Together, these findings elucidate a key metabolic pathway regulating neutrophil NETosis and highlight a targeted therapeutic strategy for treating ARDS.

## 2 Materials and Methods

### 2.1 Ethics Statement

All animal procedures were conducted in accordance with the institutional guidelines for animal care and use established by Central South University (Changsha, China) and were approved by the Institutional Review Board (IRB) of the Xiangya School of Basic Medical Sciences (Approval No. 2023‐KT026). Human neutrophils used in this study were isolated from ARDS patients at Xiangya Hospital, Central South University. All participants were formally diagnosed with ARDS and provided written informed consent for the use of clinical samples for research purposes. The study protocol involving human subjects was reviewed and approved by the Clinical Medical Ethics Committee of Xiangya Hospital, Central South University (Ethical Approval No. 2022100983, Changsha, China).

### 2.2 Mouse Model

Adult male C57BL/6J mice (8–10 weeks old, 20–25 g) were acquired from Hunan SJA Laboratory Animal Co., Ltd. (China) and housed under specific pathogen‐free conditions in the animal facility of Central South University. To induce ALI, mice were subjected to intratracheal administration of LPS (from *E. coli* O111:B4, Sigma‐Aldrich, USA, 5 mg/kg in 50 µL saline) and euthanized after 12 h. Control animals received the same volume of saline via identical delivery. The treatment group received 3‐fluoropyruvate‐loaded bovine serum albumin nanoparticles (3‐FP‐BSA NPs, 2.5 mg/kg) via intravenous injection, while the Control and ALI groups were administered BSA NPs; all treatments lasted 12 h. The in vivo dose (2.5 mg/kg) was derived from the effective in vitro concentration (200 µM) using standard body surface area conversion for mice.

### 2.3 Neutrophil Isolation From Mice

Neutrophils were isolated from either bone marrow or peripheral blood of C57BL/6J mice using immunomagnetic positive selection with anti‑lymphocyte antigen 6 complex (Ly6G) MicroBeads UltraPure (Miltenyi Biotec, USA) [23]. For bone marrow neutrophils, femurs and tibiae were flushed with ice‑cold phosphate‐buffered saline (PBS) via a 26‐gauge needle. For peripheral blood neutrophils, blood was collected via cardiac puncture, mixed with 2.0% Dextran T‐500 (Solarbio, China) in 0.9% saline at a 1.5:1 ratio for erythrocyte sedimentation, and the leukocyte‑rich supernatant was collected. After erythrocyte lysis (Solarbio), cells from both sources were labeled with Ly6G MicroBeads and positively selected using LS columns on a magnetic‐activated cell sorting MultiStand system, according to the manufacturer's instructions. Isolated Ly6G^+^ cells were resuspended in RPMI‑1640 (Gibco, USA) with 10% bovine calf serum (BCS, Gibco). Cell purity (91.98%) was routinely verified by flow cytometry and maintained under standard culture conditions (Figure S1A). For NETosis induction, neutrophils from either bone marrow or peripheral blood of mice were stimulated with 25 nM phorbol 12‑myristate 13‑acetate (PMA) [24].

### 2.4 Purity and Viability Assessment of Isolated Neutrophils

Purity and viability of isolated mouse neutrophils were assessed by Flow cytometry. Following staining with anti‐Ly6G and anti‐CD11b antibodies, neutrophil purity exceeded 92% for both bone marrow‐derived (97.51%) and peripheral blood‐derived (92.27%) populations. Viability was determined immediately after isolation using 7‐Aminoactinomycin D (7‐AAD, BioLegend, USA) staining, with viable cell percentages of 96.57% for bone marrow‐derived neutrophils and 97.58% for peripheral blood‐derived neutrophils. Cell viability was assessed by 7‐AAD staining, and the proportion of 7‐AAD^−^ viable cells was >99% before subsequent assays (Figure S4).

### 2.5 Single‐Cell RNA Sequencing Data Analysis

The single‑cell RNA‑seq dataset GSE157789, derived from peripheral blood total white blood cells of ARDS patients and healthy subjects, was downloaded from the Gene Expression Omnibus (GEO) database. Raw count matrices were processed using the Seurat package (v5) in R (v4.4.1). Cells were filtered by the following criteria: (1) number of detected genes <200 or >7000; (2) mitochondrial gene percentage >20%. After filtering, gene expression data were log‑normalized using the NormalizeData function with a scale factor of 10 000. Principal component analysis (PCA) was performed on the top 2000 highly variable genes, and the first 20 principal components were used for uniform manifold approximation and projection (UMAP) dimensionality reduction. Batch effects across different conditions were mitigated using the RunHarmony function for dataset integration. Cell clusters were identified using the FindClusters function with a resolution parameter of 0.5. Putative marker genes for each cluster were detected using the FindAllMarkers function. For pathway enrichment analysis, differentially expressed genes in neutrophils between ARDS patients and healthy controls (HCs) were subjected to Kyoto Encyclopedia of Genes and Genomes (KEGG) pathway analysis using clusterProfiler (v4.12.6) and Gene Set Enrichment Analysis (GSEA) using the fgsea package with MSigDB curated gene sets. An adjusted *P*‑value < 0.05 was considered statistically significant.

### 2.6 Targeted Metabolomics Analysis

Cells were plated in 6‐cm dishes and permitted to adhere. After attachment, the culture medium was removed, and the monolayers were washed twice with PBS. Cells were then harvested using a cell scraper, washed again with PBS, and counted. For each biological replicate, 1 × 10^7^ cells were collected by centrifugation at 2500 rpm for 5 min at 4°C. The supernatant was discarded, and cell pellets were rapidly frozen in liquid nitrogen and stored at −80°C until further processing. Targeted metabolomic profiling was performed by Majorbio (China). Metabolites were extracted by adding ice‐cold 80% (v/v) methanol containing stable isotope‐labeled internal standards to each pellet, followed by thorough vortexing to achieve complete cell lysis. After a 5‐minute incubation on ice, samples were centrifuged at 15 000×*g* for 15 min at 4°C. The resulting supernatant was collected as the metabolite extract. Chromatographic separation was performed on a SCIEX ExionLC AD UHPLC system coupled to an AB SCIEX QTRAP 7500 mass spectrometer operated in multiple reaction monitoring (MRM) mode. Raw UHPLC‐MS/MS data were processed with SCIEX OS software 3.4.5 for peak integration and quantification of target metabolites based on calibration curves. Quantification was performed using external calibration curves constructed with authentic standards, with stable isotope‐labeled internal standards used for normalization. Target metabolites were identified by comparing retention times and MRM transitions with authentic standards. Statistical and pathway enrichment analyses were conducted using MetaboAnalyst 5.0, with metabolic pathways considered significantly enriched at *p* < 0.05.

### 2.7 Seahorse XFe24 Analyzer

Mitochondrial stress tests were performed to assess the oxygen consumption rate (OCR) in neutrophils using a Seahorse XFe24 Analyzer (Agilent Seahorse Bioscience, Santa Clara, USA). Neutrophils were seeded in XFe24 plates at 30,000 cells per well and pretreated with 3‐FP or vehicle control. Before measurement, the medium was replaced with XF assay medium (Seahorse XF DMEM, pH 7.4; No.103576‐100, Agilent Seahorse Bioscience), and cells were incubated for 1 h in a non‐CO_2_ incubator. After a final wash, the sensor cartridge was calibrated, and the ATP rate assay was run using a predefined protocol in WAVE Software (v2.6.1). The mitochondrial stress test was carried out through sequential injections of oligomycin (2 µM), FCCP (1 µM), and a mixture of rotenone (0.5 µM each).

### 2.8 High‐Throughput Phosphoproteomic Profiling of Human Neutrophils

Neutrophils were isolated from human peripheral blood and stimulated with 25 nM PMA or maintained as an unstimulated control. Total protein was extracted from cell lysates using a buffer containing 8 M urea and 1% sodium dodecyl sulfate (SDS), supplemented with protease inhibitors, followed by homogenization, incubation on ice, and centrifugation. Protein concentration was determined via a bicinchoninic acid (BCA) assay (DINGGUO CHANGDHRNH, China). For each sample, 100 µg of protein was reduced with TCEP, alkylated with iodoacetamide, and acetone‐precipitated. The pellet was redissolved and digested overnight with trypsin (1:50, w/w). The resulting peptides were desalted using HLB cartridges.

Phosphopeptides were enriched from the total peptide mixture using a standardized phosphopeptide enrichment kit, based on immobilized metal affinity chromatography (IMAC) or titanium dioxide (TiO_2_) principles, following the manufacturer's protocol. The enriched phosphopeptides were then analyzed by liquid chromatography‐tandem mass spectrometry (LC‐MS/MS). Briefly, samples were separated on a C18 reversed‐phase column using a nanoflow LC system (VanquishNeo, Thermo, USA) coupled online to an Orbitrap Astral mass spectrometer (Thermo). Data were acquired in data‐independent acquisition (DIA) mode. The raw DIA data were processed using Spectronaut (v19) with the directDIA workflow against the human UniProt database. Search parameters included trypsin/P specificity, up to two missed cleavages, carbamidomethylation of cysteine as a fixed modification, and oxidation of methionine, protein N‐terminal acetylation, and phosphorylation (S/T/Y) as variable modifications. Protein and peptide false discovery rates (FDR) were set to ≤ 1%. Statistical analysis was performed using the R language. Differentially phosphorylated sites were identified based on a significance threshold of *p*‐value <0.05 (Student's *t*‐test) and a fold change >1.2 (PMA *vs*. Control). Functional annotation of proteins harboring differential phosphorylation sites was conducted via KEGG analyses. Protein‑protein interaction (PPI) network analysis was performed on the set of differentially modified proteins (fold change >1, *p* < 0.05) using the search tool for the retrieval of the Interacting Genes/Proteins database with a combined confidence score ≥ 0.7. The network was visualized in Cytoscape (v3.10.4). Gene Ontology (GO) biological process enrichment analysis was conducted on the same protein set using clusterProfiler.

### 2.9 Western Blot

Lung tissue and cultured cells were homogenized in ice‐cold radioimmunoprecipitation assay lysis buffer (Solarbio) containing protease and phosphatase inhibitor cocktails (CWBIO, China) as in our previous studies [25, 26]. Protein concentrations were determined using a BCA assay kit. Protein aliquots (25 µg per lane) were denatured at 95 °C for 10 min, resolved by SDS‐PAGE, and subsequently transferred onto polyvinylidene fluoride (PVDF) membranes (Millipore, USA). After blocking with 5% non‐fat milk or BSA for 1.5 h at room temperature, the membranes were probed with specific primary antibodies at 4 °C overnight. Following extensive washing with Tris‐buffered saline with Tween‐20, membranes were incubated with horseradish peroxidase (HRP)‐conjugated secondary antibodies for 1 h at room temperature. Protein signals were visualized using an enhanced chemiluminescence substrate and captured with an imaging system. Band intensities were quantified using Image Lab software, and antibody information is summarized in Table S1.

### 2.10 Co‐Immunoprecipitation (Co‐IP)

dHL‐60 Cells were lysed in ice‐cold Non‐Denaturing Lysis Buffer (Abbkine, KTD105‐CN) supplemented with 1× Protease Inhibitor Cocktail and 1× Phosphatase Inhibitor Cocktail to preserve phosphorylation. After centrifugation (12 000 rpm, 10 min, 4°C), protein concentrations were measured by BCA assay. Freshly prepared lysates (0.5 mg) were pre‐cleared with Protein A/G Agarose for 30 min at 4°C to reduce non‐specific binding.

For IP/Co‐IP, 40 µL of Protein A/G Agarose slurry was incubated with 2 µg of specific primary antibody (or IgG as a negative control) for 30 min at room temperature. The pre‐cleared lysates were then added and incubated for 1 h at room temperature or overnight at 4°C. After washing three times with 1× Wash Buffer, proteins were eluted with 1× SDS‐PAGE Loading Buffer at 100°C for 5 min. Samples were subjected to SDS‐PAGE and Western blotting.

### 2.11 Lactate Production

Lactate levels were measured using a commercial Lactate Assay Kit (Sigma‐Aldrich) according to the supplier's protocol. Each assay well contained a final mixture of 2 µL lactate probe, 26 µL sample, 26 µL assay buffer, and 2 µL lactate enzyme mix. Following a 30‐minute incubation at room temperature, readings were taken colorimetrically at 570 nm or fluorometrically (excitation/emission  =  535/587 nm).

### 2.12 Detection of PDHC Activity

PDHC activity was determined using a commercial assay kit (Nanjing Jiancheng Bioengineering Institute) based on the spectrophotometric detection of 2,6‐dichlorophenolindophenol (2,6‐DCPIP) reduction at 605 nm. The assay quantifies the enzyme‐catalyzed dehydrogenation of pyruvate by measuring the decrease in absorbance over a 1‐minute interval. Absorbance readings were taken at time zero (A1) and 1 min (A2), and the change in absorbance (ΔA = A1−A2) was used to calculate PDHC activity according to the manufacturer's instructions.

### 2.13 Recruitment of HCs and Clinical ARDS Patients

This study enrolled patients diagnosed with ARDS according to the Berlin definition. From March 2024 to September 2026, blood samples were obtained from 12 ARDS patients in the Intensive Care Unit of Xiangya Hospital, Central South University, and 12 age‐ and sex‐matched healthy controls (HCs) recruited from the Health Examination Center of the same hospital. Exclusion criteria included current pregnancy or lactation, age below 18 years, active hematologic or solid tumor malignancies, end‐stage organ disease, and autoimmune disorders. Written informed consent was acquired from all participants or their legal representatives. The following parameters were retrospectively collected from electronic medical records: age, sex, body mass index (BMI), PaO_2_/FiO_2_, Sequential Organ Failure Assessment (SOFA) score, Acute Physiology and Chronic Health Evaluation (APACHE) II score, white blood cell count, neutrophil count, C‐reactive protein (CRP), and procalcitonin (PCT) (Table S2).

### 2.14 Human Peripheral Blood Neutrophils

Peripheral blood samples were obtained from healthy consenting adults under an ethics‐approved protocol in accordance with the Declaration of Helsinki. Whole blood was mixed with 2.0% Dextran T‐500 (Solarbio) in 0.9% saline at a 1.5:1 ratio and allowed to settle for 30 min at room temperature. The leukocyte‐enriched supernatant was then subjected to density gradient centrifugation using a preformed Percoll (Cytiva, USA) system with 45%, 62%, and 75% layers. After spinning at 250×*g* for 30 min (without brake), neutrophils were collected from the 62%–75% interface, washed with PBS, and resuspended in RPMI 1640 containing 10% BCS. Cell purity (>88%) was evaluated by Giemsa staining before subsequent assays (Figure S1B).

### 2.15 Immunofluorescence (IF) Staining

IF staining was carried out on 3‑µm paraffin‑embedded lung sections or fixed cultured cells as previously described [27, 28]. After rinsing in PBS, samples were fixed with 4% paraformaldehyde for 15 min at room temperature and permeabilized with 0.1% Tween‑20. To reduce nonspecific background, blocking was performed for 1 h at room temperature using 10% BSA in PBS. Samples were then probed overnight at 4°C with primary antibodies, followed by extensive washing and incubation for 1 h at room temperature with appropriate fluorophore‑conjugated secondary antibodies (kept in the dark). Nuclei were counterstained with DAPI (Solarbio). Images were captured on a laser‑scanning confocal microscope (Leica SP8, Germany).

### 2.16 Cell Culture and Treatment

The human promyelocytic leukemia cell line dHL‐60 was obtained from the American Type Culture Collection (RRID: CVCL_HL60), with all cell lines confirmed to be free from contamination. Cells were cultured at 37°C in a 5% CO_2_ atmosphere using RPMI‐1640 medium supplemented with L‐glutamine, 25 mM HEPES, 10% heat‐inactivated BCS, and 1% penicillin‐streptomycin. To promote neutrophilic differentiation, dHL‐60 cells were exposed to 1 µM all‑trans retinoic acid (ATRA) combined with 1% dimethyl sulfoxide (DMSO) over 6 d. Differentiation status was assessed morphologically through Wright‐Giemsa staining. For NETosis induction, differentiated dHL‑60 cells were stimulated with 100 nM PMA [29]. To dissect the underlying mechanisms, cells were pretreated before NETosis induction as follows: with 3‑FP (200 µM) for 1 h to inhibit PDHA1, with 3‑FP‑BSA NPs at a concentration equivalent to 200 µM free 3‑FP (based on encapsulation efficiency and loading content) to enable direct comparison with the free drug, with AZD7545 (10 µM) for 4 h to activate PDHA1, with mitoQ (1 µM) for 1 h before AZD7545 exposure to activate PDHA1, or with Compound C (10 µM) for 1 h to block AMPK activity. All modulators were sourced from MedChemExpress.

### 2.17 Plasmids and Transfection

FLAG‐tagged PDHA1 mutant plasmids were generated by full‐length gene synthesis. The human PDHA1 coding sequence containing an N‐terminal FLAG tag (DYKDDDDK) and the indicated point mutations (S293A or S314A) was synthesized by Sangon Biotech Co., Ltd. (Shanghai, China). The synthesized fragments were cloned into the pcDNA3.1(+) vector using KpnI/NotI restriction sites. Recombinant plasmids were screened in E. coli and verified by Sanger sequencing. The confirmed pcDNA3.1(+)‐FLAG‐PDHA1‐S293A and pcDNA3.1(+)‐FLAG‐PDHA1‐S314A plasmids were used for subsequent experiments. The wild‑type PDHA1 expression plasmid (PDHA1‑WT) was constructed using the same backbone without the mutation.

To knock down endogenous PDHA1, a short hairpin RNA (shRNA) targeting human PDHA1 was designed and cloned into the GV248 lentiviral vector (Shanghai GeneChem Co., Ltd., China). The shRNA oligonucleotide (sequence: ccgggcCAATCAGTGGATCAAGTTTctcgagAAACTTGAT‐CCACTGATTGgctttttg) was synthesized as complementary sense and antisense strands with AgeI and EcoRI overhangs, annealed, and ligated into AgeI/EcoRI‑linearized GV248. Transfection efficiency was assessed by Western blot detection of PDHA1 S293 expression. dHL‑60 cells were transfected using Lipofectamine 3000 reagent (Invitrogen, USA) following the manufacturer's instructions with slight adjustments. Briefly, cells in the logarithmic growth phase were seeded in 24‑well plates at a density of 5 × 10^5^ cells per well in 0.5 mL of complete growth medium (IMDM containing 20% FBS, no antibiotics). For each transfection, 1.0 µg of plasmid DNA and 2 µL of P3000 reagent were diluted in 50 µL of Opti‑MEM I Reduced Serum Medium. In parallel, 2 µL of Lipofectamine 3000 reagent was diluted in 50 µL of Opti‑MEM. The diluted DNA was combined with the diluted Lipofectamine 3000 reagent, incubated at room temperature for 15 min, and added dropwise to the cells. After gentle swirling, plates were returned to a 37°C, 5% CO_2_ incubator. Following 30 min of incubation, 0.5 mL of complete medium supplemented with 20% BCS was added to each well. For transient knockdown experiments, cells were generally harvested 48 h after transfection for subsequent analysis.

### 2.18 RNA Extraction and Real‐Time Quantitative Polymerase Chain Reaction (Real‐Time PCR)

Total RNA was extracted from samples using RNAiso Plus (Takara, Japan) and assessed for purity/concentration by spectrophotometry (NanoDrop, Thermo Fisher Scientific) as previously described [30, 31]. RNA integrity was verified via 1% agarose gel electrophoresis. Genomic DNA was removed and cDNA synthesized from 1 µg total RNA using PrimeScript RT Reagent Kit with gDNA Eraser (Takara, RR047A). RT‐qPCR was performed in triplicate with TB Green Premix Ex Taq II (Takara, RR420A) on a CFX96 Touch system (Bio‐Rad Laboratories Ltd., UK). Amplification conditions: 95°C 30 s, 40 cycles of 95°C 5 s and 60°C 30 s. Relative mRNA expression was calculated by the 2^−ΔΔCt^ method with β‐actin as endogenous control. Primer sequences are listed in Table S3.

### 2.19 RNA Sequencing

To characterize the transcriptional response of HL‐60 cells to PMA stimulation, total RNA was extracted using RNAiso Plus reagent (Takara) and processed for sequencing on an Illumina NovaSeq 6000 platform at Majorbio Technology (China). Raw reads in FASTQ format underwent quality control with FASTQC, followed by removal of low‐quality sequences using FASTX‐Toolkit. High‐quality reads were aligned to the human reference genome, Genome Reference Consortium Human Build 38, with TopHat, guided by gene annotation in GTF format. Gene expression levels were quantified as fragments per kilobase of transcript per million mapped reads (FPKM), and read counts were generated using high‐throughput sequence analysis in Python (HTSeq). Differential expression analysis between control and PMA‐treated samples was performed using the DESeq2 package in R, with genes with an adjusted *p* < 0.05 as statistically significant. All analyses were conducted on the Majorbio Cloud Platform (www.majorbio.com).

### 2.20 Preparations of 3‐FP‐BSA NPs

3‐FP‐BSA NPs were synthesized using a desolvation method. Briefly, 20 mg of BSA was dissolved in 1 mL of ultrapure water, and 2 mg of 3‐FP was separately dissolved in 3.5 mL of ethanol. The aqueous BSA solution was vigorously stirred at 25°C, and the ethanolic 3‐FP solution (0.23 mg/mL) was injected at a rate of 2 mL/min to induce colloidal nanoparticle formation. After 15 min of continuous stirring, 100 µL of 2% glutaraldehyde solution was added dropwise to cross‐link the nanoparticles. The reaction was allowed to proceed for 16 h, after which the resulting 3‐FP‐BSA NPs were collected by centrifugation, washed, and resuspended in PBS for subsequent experiments.

### 2.21 Transmission Electron Microscopy (TEM)

Samples were fixed in 2.5% glutaraldehyde at 4°C overnight, post‐fixed with 1% osmium tetroxide for 2 h, and then dehydrated through a graded ethanol series. The specimens were subsequently embedded in epoxy resin. Ultrathin sections (70 nm) were cut using an ultramicrotome, stained with uranyl acetate and lead citrate, and examined under a transmission electron microscope (JEOL JEM‐1400, Japan). Images were captured with a digital camera system.

### 2.22 Characterization of 3‐FP‐BSA NPs

The morphology of the nanoparticles was examined by transmission electron microscopy (JEOL‐JEM‐2100Plus, Japan). Mean particle size and zeta potential were assessed using a Zetasizer Nano ZS instrument (Malvern, UK). Nanoparticle stability was evaluated under simulated physiological conditions in vitro. The encapsulation efficiency (EE%) of 3‐FP in BSA NPs was quantified via size exclusion chromatography coupled with LC‐MS. Cytotoxicity profiles of different formulations were determined using a lactate dehydrogenase (LDH) release assay to measure cell viability.

### 2.23 Release of 3‐FP‐BSA NPs

The release profile of 3‐FP‐BSA NPs was determined by the dialysis method. 2 mL of 3‐FP and 3‐FP‐BSA NPs were put into a dialysis bag (molecular weight of 14 kDa). The samples were submerged in 40 mL of PBS (pH 7.4) and incubated at 37°C in a thermostatic shaking incubator (100 r/min). 1 mL of dialysate was taken and replaced with 1 mL of constant‐temperature PBS at 0.5, 1, 2, 4, 6, 8, 12, 24, and 48 h. The amount of 3‐FP released from 3‐FP‐BSA NPs was quantified with a microplate reader (Bio‐Rad). The percentage of 3‐FP released from 3‐FP‐BSA NPs was plotted versus time.

### 2.24 Hemolytic Assay

The hemocompatibility of BSA‐NPs and 3‐FP‐BSA‐NPs was evaluated using a hemolysis assay. Erythrocytes isolated from C57BL/6J mouse blood were resuspended in PBS to a final concentration of 2% (v/v). Test samples were supplemented with either BSA NPs or 3‐FP‐BSA NPs, while deionized water and PBS served as positive and negative controls, respectively. After incubation at 37°C for 4 h with gentle shaking, the samples were centrifuged at 1500 rpm for 10 min. Hemoglobin release was determined by measuring the absorbance of the supernatant at 540 nm. The hemolysis percentage was calculated as follows: Hemolysis (%) = [(A_sample‐A_negative)/(A_positive‐A_negative)] ×100%, where A_sample, A_negative, and A_positive represent the absorbance values of the test sample, negative control, and positive control, respectively.

### 2.25 Hematoxylin and Eosin (H&E) Staining

Tissue specimens were fixed in 4% paraformaldehyde, embedded in paraffin, and sectioned as in our previous study [32]. Slices were then incubated at 64°C for 1 h, deparaffinized, and dehydrated. Subsequent H&E staining was performed following a standard protocol, including steps for hematoxylin counterstaining, eosin staining, clearing, and coverslipping. Finally, whole‐slide images were acquired using a digital slide scanner.

### 2.26 Determination of Supernatant dsDNA

The PicoGreen reagent was freshly diluted 1:200 in Tris‐ethylenediaminetetraacetic acid (TE) buffer (10 mM Tris‐HCl, 1 mM ethylenediaminetetraacetic acid, pH 7.5). A standard curve was prepared using lambda DNA at concentrations ranging from 0 to 2 µg/mL in TE buffer. All standards and samples were analyzed in duplicate by combining 50 µL of each with an equal volume of the diluted PicoGreen working solution in black 96‐well plates with clear bottoms. After a 10‐minute incubation at room temperature in the dark, fluorescence was measured using a microplate reader (excitation: 480 nm, emission: 520 nm).

### 2.27 Enzyme‐Linked Immunosorbent Assay (ELISA)

Concentrations of tumor necrosis factor‐α (TNF‐α) were measured using commercial ELISA kits (Invitrogen; Thermo Fisher Scientific) according to the manufacturer's protocols. Likewise, MPO‐dsDNA and citH3‐dsDNA complexes were evaluated using specific ELISA kits (JONLNBIO, China) following the vendor's instructions. Optical density recorded at 450 nm was converted to concentration values based on a calibrated standard curve. All cytokine measurements were normalized to total protein content, which was determined via a BCA protein assay kit.

### 2.28 Reactive Oxygen Species (ROS) Detection

Mitochondrial ROS generation was assessed using MitoSOX Red (MedChemExpress). A 1 µM working solution was prepared, and 500 µL was added to each well. After incubation at 37°C for 30 min, cells were washed three times with PBS and imaged using a fluorescence microscope (Nikon Eclipse Ti2, Japan).

### 2.29 Mitochondrial Membrane Potential (MMP) Assay

MMP was assessed using the fluorescent probe JC‐1 (ACRO Biosystems, China). Differentiated HL‐60 cells were collected and incubated with JC‐1 at 37°C for 20 min, followed by imaging with confocal laser scanning microscopy. The MMP was represented by the ratio of red (aggregates) to green (monomers) fluorescence intensity.

### 2.30 LDH Activity

LDH activity in cell culture supernatants was measured using a commercial LDH Cytotoxicity Assay Kit (Nanjing Jiancheng Bioengineering Institute, China). This assay quantifies LDH based on the enzymatic conversion of lactate to pyruvate coupled with NAD^+^ reduction to nicotinamide adenine dinucleotide (NADH). Following the indicated treatments, LDH activity was determined according to the manufacturer's instructions, and absorbance was read at 450 nm using a microplate reader.

### 2.31 Statistical Analysis

All quantitative measurements were done at least in triplicate, and the mean ± SD was presented. Comparison of the mean values between different groups was performed by ANOVA (Dunnett's test) or two‐tailed unpaired *t*‐test using GraphPad software 9.0.1 (San Diego, CA, USA). A *p*‐value ≤ 0.05 was considered to be statistically significant.

## 3 Results

### 3.1 Neutrophils Undergo Selective Metabolic Reprogramming Toward OXPHOS During ALI

Single‑cell RNA‑seq analysis of peripheral blood samples from ARDS patients (GSE157789) revealed a significant expansion of neutrophils among the seven major immune cell subsets identified (Figure 1A). To rigorously define the cell population annotated as neutrophils, we analyzed the expression of canonical marker genes, specifically C‐X‐C motif chemokine receptor 2 (CXCR2), Fc gamma receptor IIIb (FCGR3B), and colony‐stimulating factor 3 receptor (CSF3R). We confirmed their high and specific expression in the neutrophil cluster (Figure S1A–D). Furthermore, KEGG pathway enrichment analysis and GSEA of transcriptomic data revealed that OXPHOS, pyruvate metabolism, and hypoxia‐inducible factor 1α (HIF‐1α)‐related glycolysis signaling were significantly activated in neutrophils from ARDS patients (Figure 1B,C). Consistently, analysis of an independent scRNA‑seq dataset derived from lung tissue neutrophils (GSE223793) also demonstrated a significant enrichment of the OXPHOS pathway (Figure S2A,B). To examine metabolic alterations during NETosis, peripheral blood neutrophils were first isolated from human donors and subsequently stimulated with PMA. Subsequent targeted metabolomics profiling revealed substantial changes in intermediate metabolite levels in neutrophils undergoing NETosis compared with the Control. Principal component analysis (PCA) clearly separated the NETosis group from the control group, confirming distinct metabolic profiles between the two conditions. Volcano plot analysis identified a total of 21 differentially accumulated metabolites, of which 9 were upregulated and 12 were downregulated (Figure S3A,B). KEGG pathway analysis further supported this metabolic rewiring, highlighting marked perturbations in OXPHOS and glucose metabolism (Figure 1D). Notably, tricarboxylic acid (TCA) cycle intermediates accumulated while glycolytic metabolites decreased (Figure 1E). Metabolically, peripheral blood neutrophils from ALI mice undergoing NETosis exhibited enhanced OXPHOS, demonstrated by elevated OCR (Figure 1F,G), maximal respiration (Figure 1H), and ATP‐linked respiration (Figure 1I). Consistent with these bioenergetic changes, Western blot analysis confirmed the upregulation of key OXPHOS components (Figure 1J,K). Consistent with these findings, neutrophils from ARDS patients also showed increased expression of OXPHOS components (Figure S2C‐F). NETosis was accompanied by partially reduced lactate production in neutrophils from both ALI mice (Figure 1L) and ARDS patients (Figure S2G), while basal lactate levels remained maintained, suggesting that glycolysis remains active but with a redistribution of metabolic flux. Together, these results indicate that neutrophils undergoing NETosis exhibit a selective metabolic rewiring in which pyruvate is redirected from lactate production toward mitochondrial oxidation during ALI.

**FIGURE 1:**
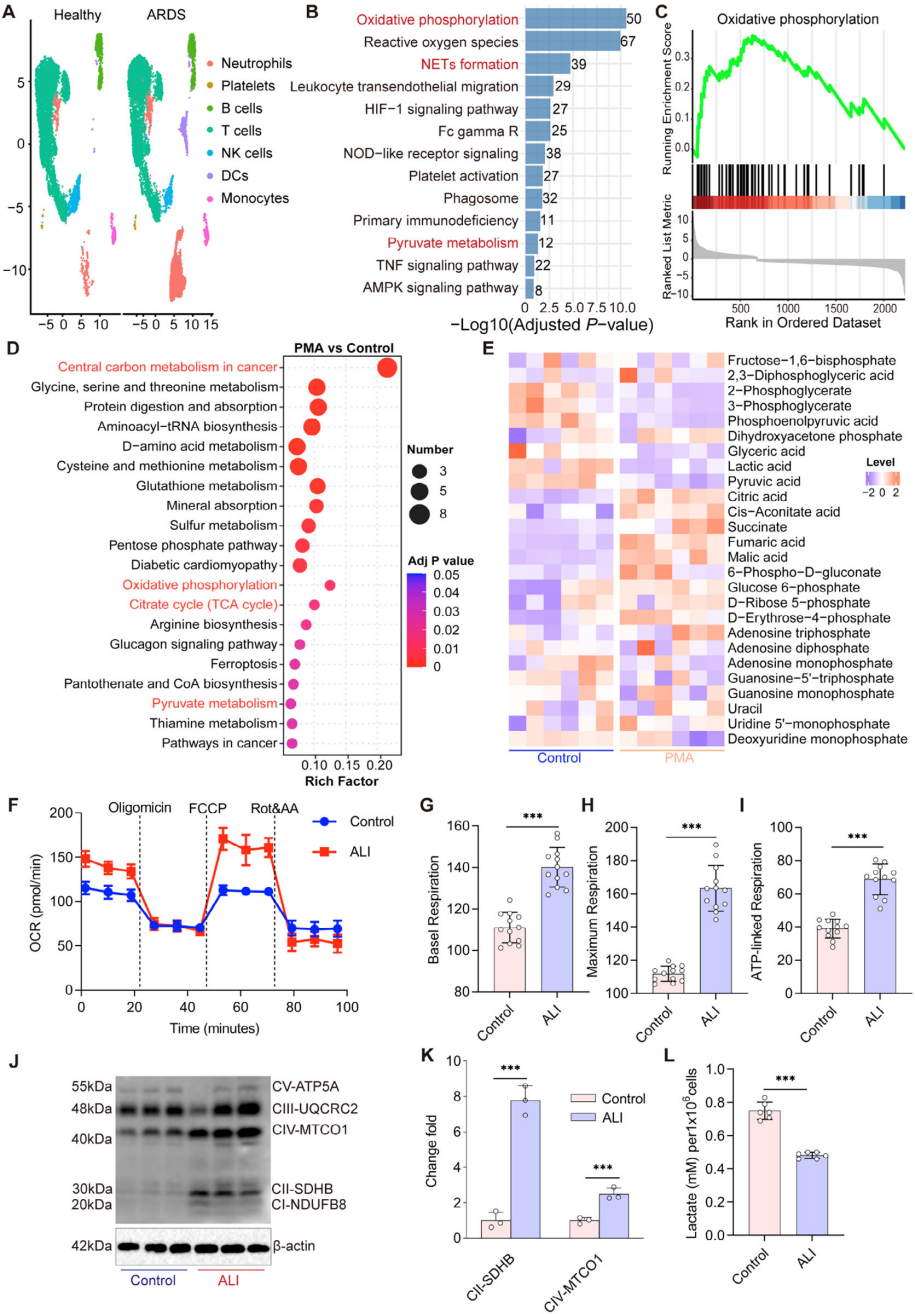
Enhanced OXPHOS and altered glucose metabolism in neutrophils during NETosis and ALI. (A) Distribution of 7 subtypes of cells by UMAP, colored by cell types (GSE157789). (B) KEGG pathway enrichment analysis was performed on the neutrophils. (C) GSEA of transcriptomic data revealed that OXPHOS signaling was significantly activated in neutrophils from ARDS patients. (D) Heatmap showing targeted metabolomic profiles in PMA‑treated human peripheral blood neutrophils, *n* = 6. (E) Targeted metabolomics analysis depicting top metabolomic pathways enriched in PMA‑treated human peripheral blood neutrophils, *n* = 6. (F,G) OCR was obtained using a Seahorse XFe24 Analyzer. (H,I) Statistical analyses of maximal respiration and ATP‐linked respiration in peripheral blood neutrophils from ALI mice, *n* = 12. (J,K) OXPHOS‐related proteins: ATP5A, MTCO1, UQCRC2, SDHB, and NDUFB8 in peripheral blood neutrophils from ALI and Control mice were detected by Western blot, *n* = 3. (L) Analysis of lactate production in peripheral blood neutrophils from ALI mice, *n* = 6. Student's *t*‐test (two‐tailed, unpaired) was used to compare Control and ALI. ***p < 0.001.

### 3.2 Selective Reduction of PDHA1 Phosphorylation at the Inhibitory Ser293 Site During Neutrophil NETosis

Pyruvate occupies a central position in cellular metabolism, bridging glycolysis and the TCA cycle. Its metabolic fate is governed by the balance between two key enzymes: LDHA, which reduces pyruvate to lactate to maintain glycolytic flux, and PDHC, which channels carbon into the TCA cycle via oxidative decarboxylation to acetyl‑CoA [33]. Within this pathway, PDHA1, the catalytic subunit of PDHC, emerged as a key protein of particular interest [34]. We first recruited patients with ARDS along with HCs and observed that PDHC activity was significantly elevated in peripheral blood neutrophils from ARDS patients (Figure 2A). To investigate whether this altered PDHC activity is linked to NETosis, and given that PDHA1 activity is regulated by phosphorylation [35], we performed phosphoproteomic profiling of PMA‐stimulated human neutrophils. Compared with controls, 895 phosphorylation sites were significantly upregulated and 985 sites downregulated in NETotic neutrophils (Figure 2B,C). We mapped the differentially expressed phosphoproteins to the Interactive Pathways Explore (IPATH) metabolic pathway diagram and observed a pronounced enhancement in the TCA cycle (Figure 2D). GO enrichment network analysis revealed that PDHA1 underwent significant differential modification within the enriched metabolic pathways (Figure 2E). There were 4 phosphorylation sites of PDHA1 containing with quantitative information. However, only the inhibitory Ser293 site showed a pronounced reduction (Figure 2F,G). These results suggest that the reduction of PDHA1 phosphorylation at Ser293 plays an essential role in neutrophil NETosis.

**FIGURE 2:**
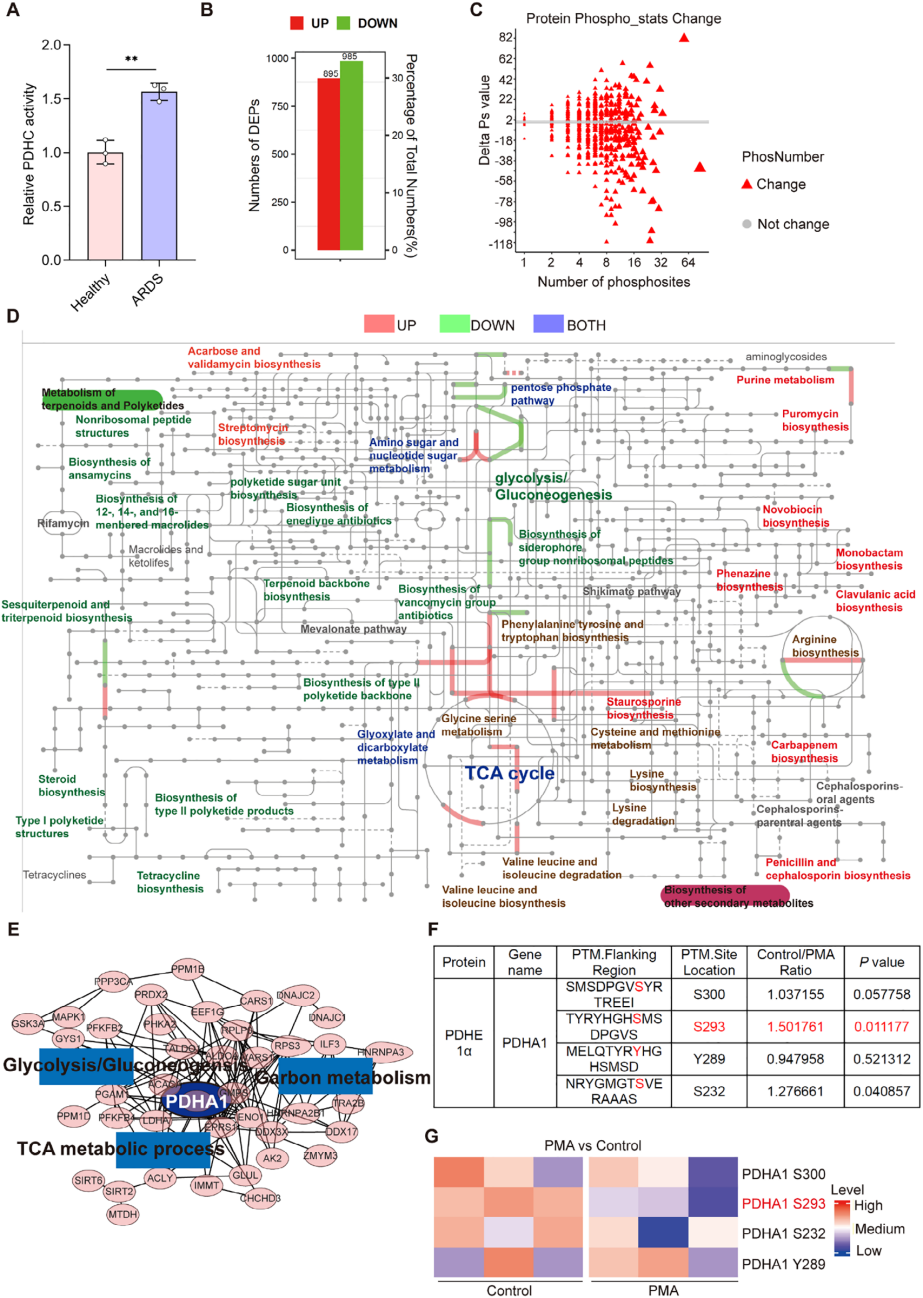
Selective reduction of PDHA1 phosphorylation at Ser293 during neutrophil NETosis. (A) Relative PDHC activity in peripheral blood neutrophils isolated from ARDS patients and HCs (*n* = 3). Phosphoproteomic profiling revealed global phosphorylation changes in PMA‐stimulated human peripheral blood neutrophils. (B) The number and percentage distribution of up‐regulated and down‐regulated differentially modified proteins. (C) Relationship between the alteration in phosphorylation and the number of phosphosites on the corresponding proteins. (D) The IPATH metabolic network diagram showing differentially enriched pathways. (E) PPI network and GO biological process enrichment of differentially modified phosphoproteins identified in PMA‑stimulated versus control human peripheral blood neutrophils. (F) PDHA1 exhibited differential phosphorylation across specific residues. (G) The phosphorylation level at PDHA1 S300, S293, S232, and Y289 was determined in PMA‐treated neutrophils by Phosphorylation omics, *n* = 3. Student's *t*‐test (two‐tailed, unpaired) was used. **p < 0.01.

### 3.3 PDHA1 Dephosphorylation at Ser293 is Associated With Neutrophil NETosis

Given that dephosphorylation at Ser293 enhances PDHA1 enzymatic activity [36], we next validated these findings in clinical and experimental models. A total of 12 ARDS patients were enrolled, comprising an initial discovery cohort (*n* = 6, Figure 3B,C) and an independent validation cohort (*n* = 6, Figure S2C,D); healthy controls (*n* = 6 per cohort) were age‐ and sex‐matched. In peripheral blood neutrophils from ARDS patients, PDHA1 phosphorylation at Ser293 was significantly decreased, with no notable changes at other phosphosites. This reduction coincided with elevated levels of the NETosis markers citH3 and MPO (Figure 3B–D). IF staining further corroborated this pattern, showing reduced PDHA1 p‑Ser293 together with increased NETosis marker expression (Figure 3E). Similarly, in an LPS‐induced murine model of ALI, peripheral blood neutrophils exhibited a specific reduction in Ser293 phosphorylation without alterations at other sites (Figure 3F–H). This decrease correlated inversely with the expression of NETosis‐related proteins (Figure 3G–H). IF staining in ALI mice peripheral blood neutrophils demonstrated coordinated elevation of NETosis markers and reduction in PDHA1 p‑Ser293 (Figure 3I,J). Collectively, these results demonstrate that enhanced PDHC activity, mediated through specific PDHA1 dephosphorylation at Ser293, is associated with neutrophil NETosis during ALI/ARDS.

**FIGURE 3:**
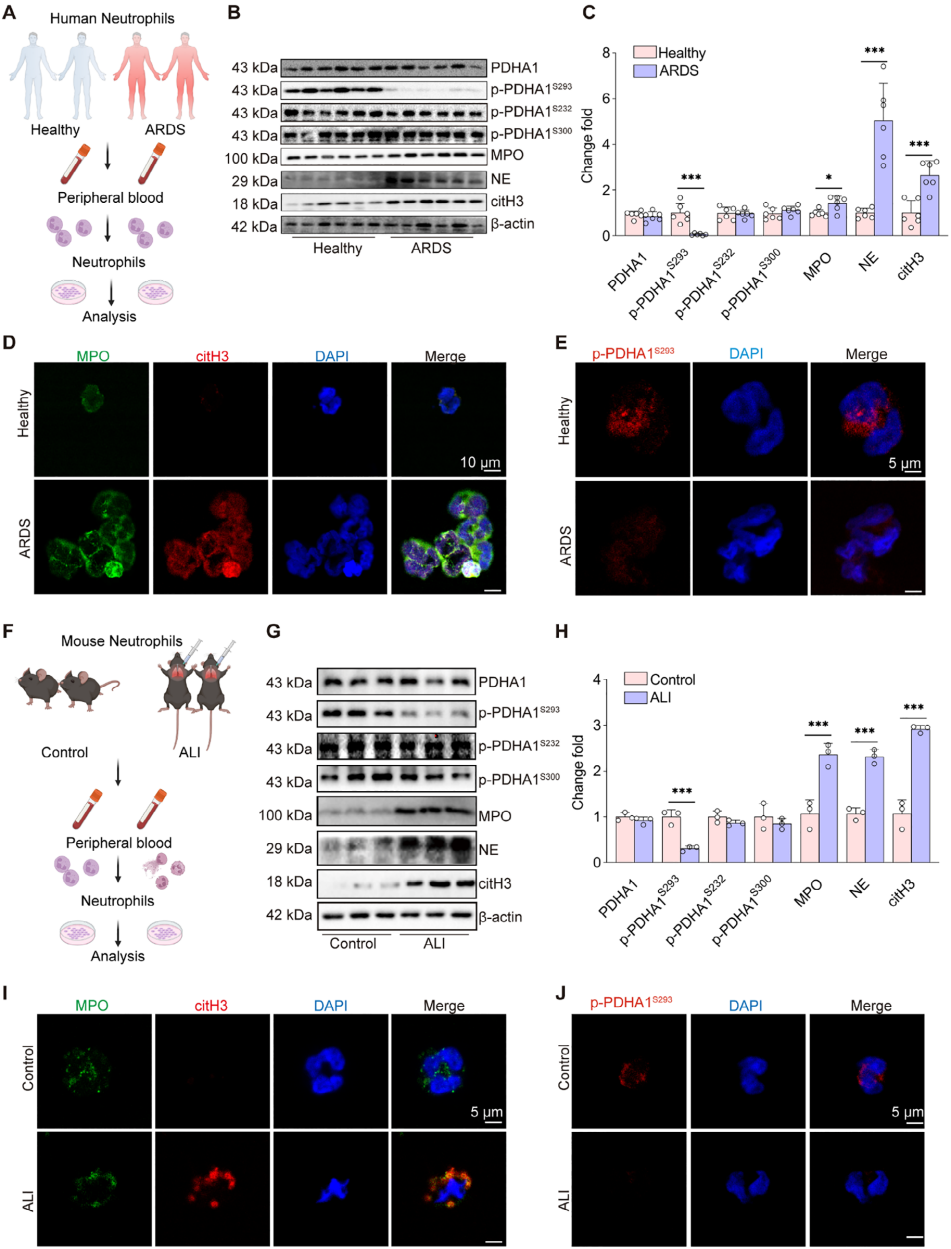
PDHA1 hyperactivity in neutrophils is positively associated with enhanced NETosis. (A) Schematic overview of experimental design for B to E. (B,C) Western blot was used to detect PDHA1 S293, S232, S300, MPO, NE, and citH3 levels in peripheral blood neutrophils isolated from ARDS patients, *n* = 6. (D) The co‐localization of MPO and citH3 was assessed by IF in peripheral blood neutrophils isolated from ARDS patients; scale bar = 10 µm. (E) IF was used to detect p‐PDHA1 S293 levels in peripheral blood neutrophils isolated from ARDS patients; scale bar = 5 µm. (F) Schematic overview of the experimental design for G to J. (G,H) Western blot was used to detect p‐PDHA1 S293, S232, S300, MPO, NE, and citH3 expression in peripheral blood neutrophils isolated from ALI mice, *n* = 3. (I) The co‐localization of MPO and citH3 was assessed by IF in peripheral blood neutrophils isolated from ALI mice; scale bar = 5 µm. (J) IF was used to detect PDHA1 S293 expression in peripheral blood neutrophils isolated from ALI mice; scale bar = 5 µm. Statistical significance was determined by Student's *t*‐test (two‐tailed, unpaired). *p < 0.05 and ***p < 0.001.

### 3.4 PDHA1 Dephosphorylation at Ser293 Drives Selective Metabolic Reprogramming, Promoting NETosis

Our findings indicate that reduced PDHA1 phosphorylation at Ser293 is consistently linked to NETosis during ALI/ARDS. We generated a phosphodeficient PDHA1 mutant (S293A) to mimic constitutive activation of PDHA1. Overexpression of a phosphodeficient PDHA1‐S293A mutant enhanced PDHC activity (Figure 4A) and promoted a selective metabolic shift characterized by enhanced OXPHOS and limited glycolytic activity. PDHA1‐S293A mutant enhanced mitochondrial function in dHL‐60 cells, as evidenced by increased OCR, ATP‐linked respiration, and maximal respiration (Figure 4B–E). Consistent with these functional improvements, the PDHA1‐S293A mutant upregulated the expression of OXPHOS‐related proteins, including ATP synthase F1 subunit alpha (ATP5A), MTCO1, SDHB, and cytochrome b‐c1 complex subunit 2 (NDUFB8) in dHL‐60 cells (Figure 4F,G), while it concurrently decreased lactate production in NETosis neutrophils (Figure 4H). Collectively, these findings indicate that PDHA1 hyperactivation, resulting from reduced Ser293 phosphorylation, initiates a selective metabolic reprogramming in dHL‐60 cells. Furthermore, the PDHA1‐S293A mutant induced NETosis formation, as demonstrated by the upregulation of key NETosis‐associated proteins, including MPO, peptidyl arginine deiminase 4 (PAD4), NE, and citH3 (Figure 4I,J). IF analysis further confirmed the induction of MPO and citH3 (Figure 4K). The PDHA1‐S293A mutant led to a marked increase in supernatant dsDNA levels in dHL‐60 cells (Figure 4L). Collectively, these results indicate that PDHA1 hyperactivation induced by dephosphorylation at Ser293 diverts the metabolic program from glycolysis toward OXPHOS, consequently driving NETosis.

**FIGURE 4:**
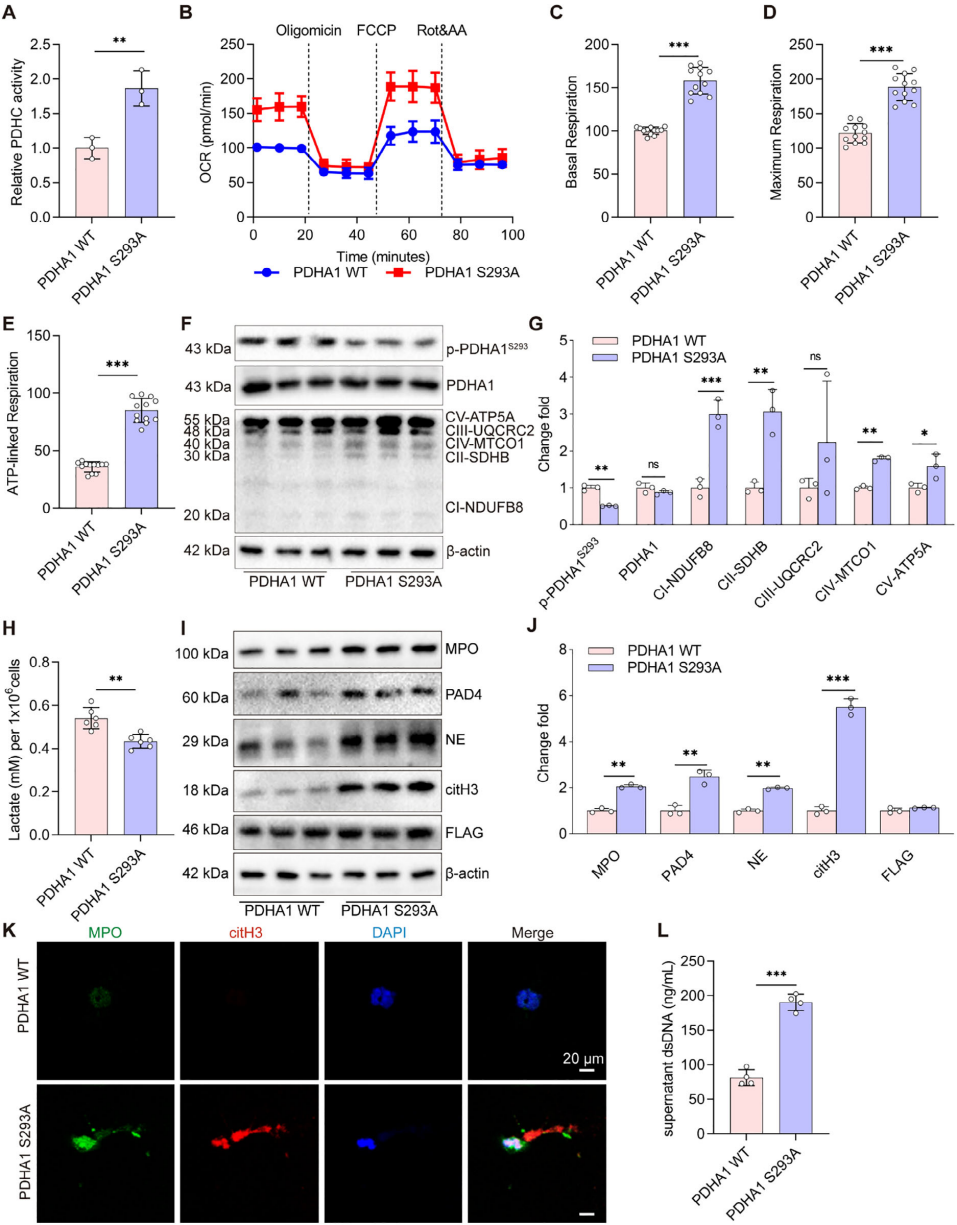
PDHA1 dephosphorylation at Ser293 drives OXPHOS and exacerbates neutrophil NETosis. dHL‐60 cells were co‐transfected with wild‐type PDHA1 WT or PDHA1‐S293A mutant. (A) The PDHA1‐S293A mutation increased PDHC activity in dHL‐60 cells. (B–E) The OCR of dHL‐60 cells transfected with the PDHA1‐WT or PDHA1‐S293A mutant was measured using a Seahorse XFe24 Analyzer (B), with statistical analyses of basal respiration (C), maximal respiration (D), and ATP‐linked respiration (E). (F,G) The expression of OXPHOS‐related proteins in dHL‐60 cells transfected with the PDHA1‐S293A mutant was detected by Western blot. (H) Analysis of lactate production in dHL‐60 cells transfected with the PDHA1‐S293A mutant. (I,J) MPO, NE, PAD4, and citH3 protein levels in dHL‐60 cells transfected with PDHA1‐WT or PDHA1‐S293A were detected by Western blot. (K) Representative IF images of MPO (green), citH3 (red), and DAPI (blue) in dHL‐60 cells; scale bar = 20 µm. (L) Measurement of extracellular dsDNA levels in the culture supernatant of dHL‐60 cells, *n* = 3. Student's *t*‐test (two‐tailed, unpaired) was used to compare PDHA1 WT and PDHA1‐S293A. *p < 0.05, **p < 0.01, and ***p < 0.001.

### 3.5 Inhibition of PDHA1 Curtails NETosis by Modulating Glucose Metabolism Reprogramming in Neutrophils

To investigate whether PDHA1 hyperactivation drives NETosis, we employed 3‐FP, a competitive inhibitor of PDHA1 [37]. Treatment with 3‐FP successfully decreased PDHC activity in PMA‐stimulated mouse bone marrow‐derived neutrophils (Figure 5A). PDHA1 inhibition attenuated the PMA‐induced increases in overall OCR (Figure 5B), as well as basal respiration (Figure 5C), maximal respiration (Figure 5D), and ATP‐linked respiration (Figure 5E). Consistent with these functional alterations, Western blot analysis further demonstrated that the PMA‐induced upregulation of OXPHOS‐related proteins was reversed upon 3‐FP treatment (Figure 5F,G). Concomitantly, PDHA1 inhibition enhanced PMA‐induced lactate production (Figure 5H), collectively indicating that PDHA1 is critical for shifting metabolism toward enhanced OXPHOS during neutrophil NETosis. Furthermore, scanning electron microscopy results revealed abundant extracellular net‐like structures surrounding PMA‐stimulated mouse bone marrow‐derived neutrophils, which were markedly diminished in PDHA1 inhibition (Figure 5I). Consistent with this structural observation, 3‐FP treatment reduced the PMA‐induced upregulation of NETosis markers (MPO, NE, PAD4, and citH3) at the protein level (Figure 5J,K). IF analysis further confirmed the reduction in MPO and citH3 (Figure 5L). Similarly, inhibition of PDHA1 led to a marked decrease in supernatant dsDNA level in PMA‑treated mouse bone marrow‐derived neutrophils (Figure 5M). In line with the pharmacological results, *Pdha1* shRNA also suppressed PMA‐induced NETosis in dHL‐60 cells, as evidenced by reduced expression of NETosis‐related markers (MPO, NE, citH3, and PAD4) (Figure S5A–C) and decreased supernatant levels of MPO‐dsDNA and dsDNA (Figure S5D,F). PMA‐treated neutrophils transfected with *Pdha1* shRNA exhibited a ballooning (bubble‐like) morphology and reduced plasma membrane rupture (Figure S5E). Collectively, these data demonstrate that suppressing the PDHA1‐mediated shift toward oxidative glucose metabolism attenuates NETosis, suggesting that PDHA1 hyperactivity‐dependent selective metabolic reprogramming acts as a key driver of NETosis in neutrophils.

**FIGURE 5:**
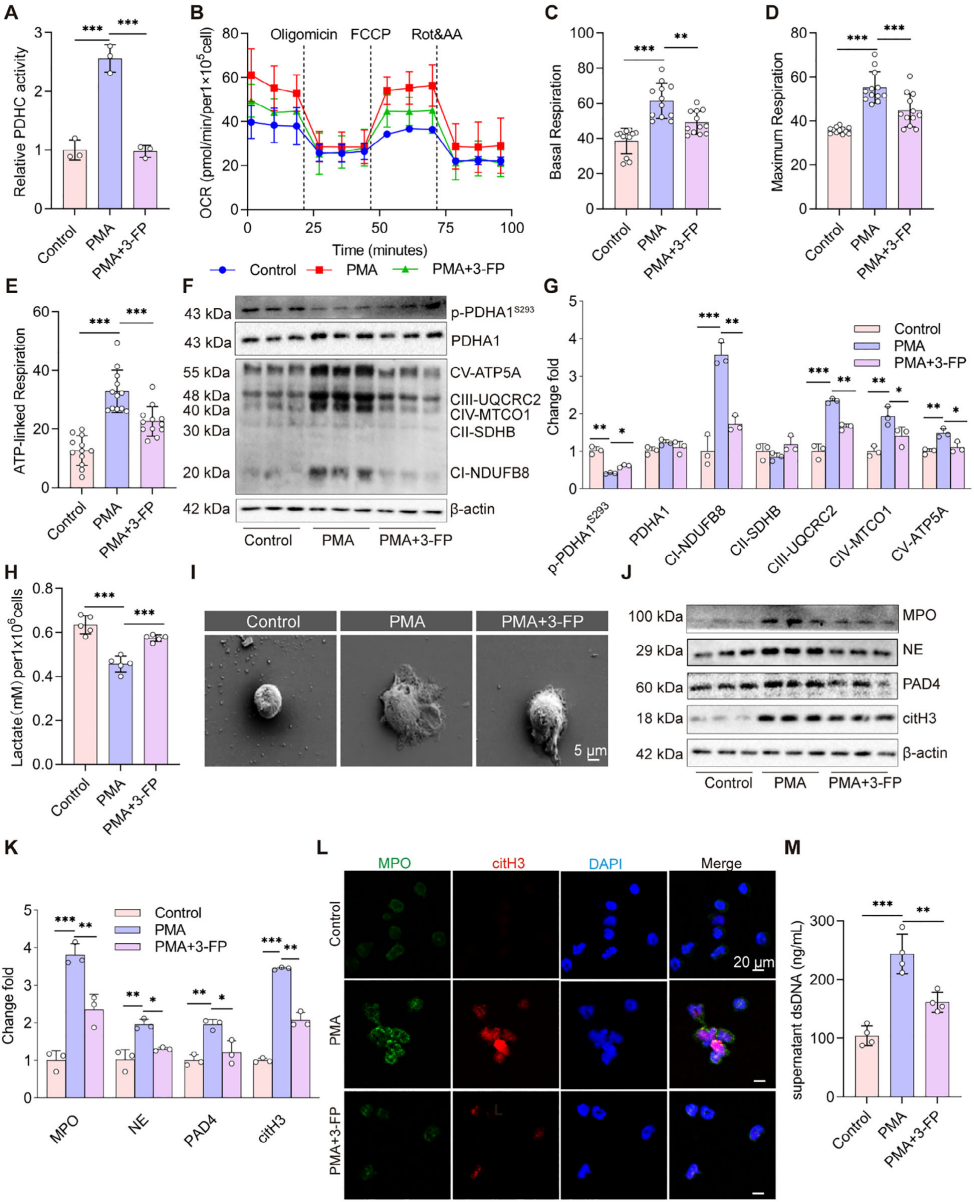
Inhibition of PDHA1 attenuates neutrophil NETosis. Mouse bone marrow‐derived neutrophils were exposed to PDHA1 competitive inhibitor 3‐FP before PMA administration. (A) PDHC activity was measured by a commercially available PDHC activity assay kit, n = 3. (B,C) OCR was obtained using a Seahorse XFe24 Analyzer. (D,E) Statistical analyses of maximal respiration and ATP‐linked respiration in treated mouse bone marrow‐derived neutrophils. (F,G) OXPHOS‐related proteins: ATP5A, MTCO1, UQCRC2, SDHB, and NDUFB8 were detected by Western blot. (H) Analysis of lactate production. (I) Scanning electron microscopy images depict the morphological features of neutrophils treated with either Control or 3‐FP with PMA stimulation, scale bar = 5 µm. (J,K) Western blot for MPO, NE, PAD4, and citH3 in PMA‐treated mouse bone marrow‐derived neutrophils, *n* = 3. (L) IF for MPO and citH3 protein expression in PMA‐induced NETosis with or without 3‐FP treatment, *n* = 3, scale bar = 20 µm. (M) The level of dsDNA content in the supernatant, *n* = 4. Statistical significance was determined by one‐way ANOVA. *p < 0.05, **p < 0.01, and ***p < 0.001.

### 3.6 Neutrophil‑Targeted 3‐FP‐BSA NPs Suppress NETosis In Vitro

To investigate the function of PDHA1 on neutrophils in orchestrating ALI inflammation, neutrophil‐specific PDHA1‐inhibited nanoparticles were generated. The synthetic route of 3‐FP‐BSA NPs is shown in Figure S6A, including three previously reported steps [38, 39]. Denatured BSA nanoparticles, prepared via techniques like ethanol desolvation, are selectively internalized by activated, but not resting, neutrophils, enabling targeted drug delivery to inflamed tissue. This specificity arises because Fcγ receptors (primarily FcγRIII/CD16b) on adherent neutrophils recognize the denatured albumin as an immune‐complex‐like structure [39, 40]. Following uptake, nanoparticle‐loaded neutrophils migrate across the vascular endothelium, serving as active carriers that precisely transport drugs to disease sites while minimizing off‐target effects and maintaining the native function of circulating neutrophils [41]. The TEM image depicted the three‐layer core‐shell structure of the 3‐FP‐BSA‐NPs with polydopamine coating (Figure 6A). Both nanoparticles had a similar particle size of 293.9 ± 5.1 nm (Figure 6A). The zeta potential was −43.73 ± 1.3 mV for BSA NPs and −51.07 ± 3.5 mV for 3‐FP‐BSA NPs (Figure 6B). Moreover, the cumulative release of 3‐FP‐BSA‐NPs reached 76% within 48 h (Figure 6C). These results indicated that 3‐FP‐BSA NPs possessed a uniform size, favorable morphology, and a sustained 3‐FP release profile. Cytocompatibility was confirmed by the lack of significant LDH release (Figure 6D) and the absence of hemoglobin leakage (Figure S6B,C) following treatment with 3‐FP‐BSA‐NPs, demonstrating their high biocompatibility. The capacity of 3‐FP‐BSA‐NPs to target NETs was further investigated. Previous studies have demonstrated that BSA‐modified nanoparticles could be located by neutrophils and accumulated within the lesion site [41]. Hence, BSA was employed as a specific Fcγ receptor to enhance the nanoparticle accumulation in neutrophils. First, the targeting ability of BSA was evaluated using Cy5‐labeled nanoparticles (3‐FP‐BSA‐NP@cy5). Confocal microscopy revealed that bone marrow‐derived neutrophils could effectively internalize the 3‐FP‐BSA‐NPs both in vitro (Figure 6E) and within the lungs of LPS‐induced ALI mice (Figure 6F,G), in contrast to the negligible uptake observed in macrophages (Figure S6D). Then, the ability of the nanoparticles to inhibit NETosis was further investigated. As expected, 3‐FP‐BSA‐NP treatment significantly reduced PDHC activity in PMA‐stimulated bone marrow‐derived neutrophils (Figure 6H). Consistently, it also effectively suppressed the levels of key NETosis markers MPO, NE, PAD4, and citH3 (Figure 6I–K) and dsDNA in the supernatants after PMA stimulation (Figure S6E). These findings indicate that 3‐FP‐BSA NPs possess effective neutrophil targeting capability and a superior capacity to inhibit NETosis formation.

**FIGURE 6:**
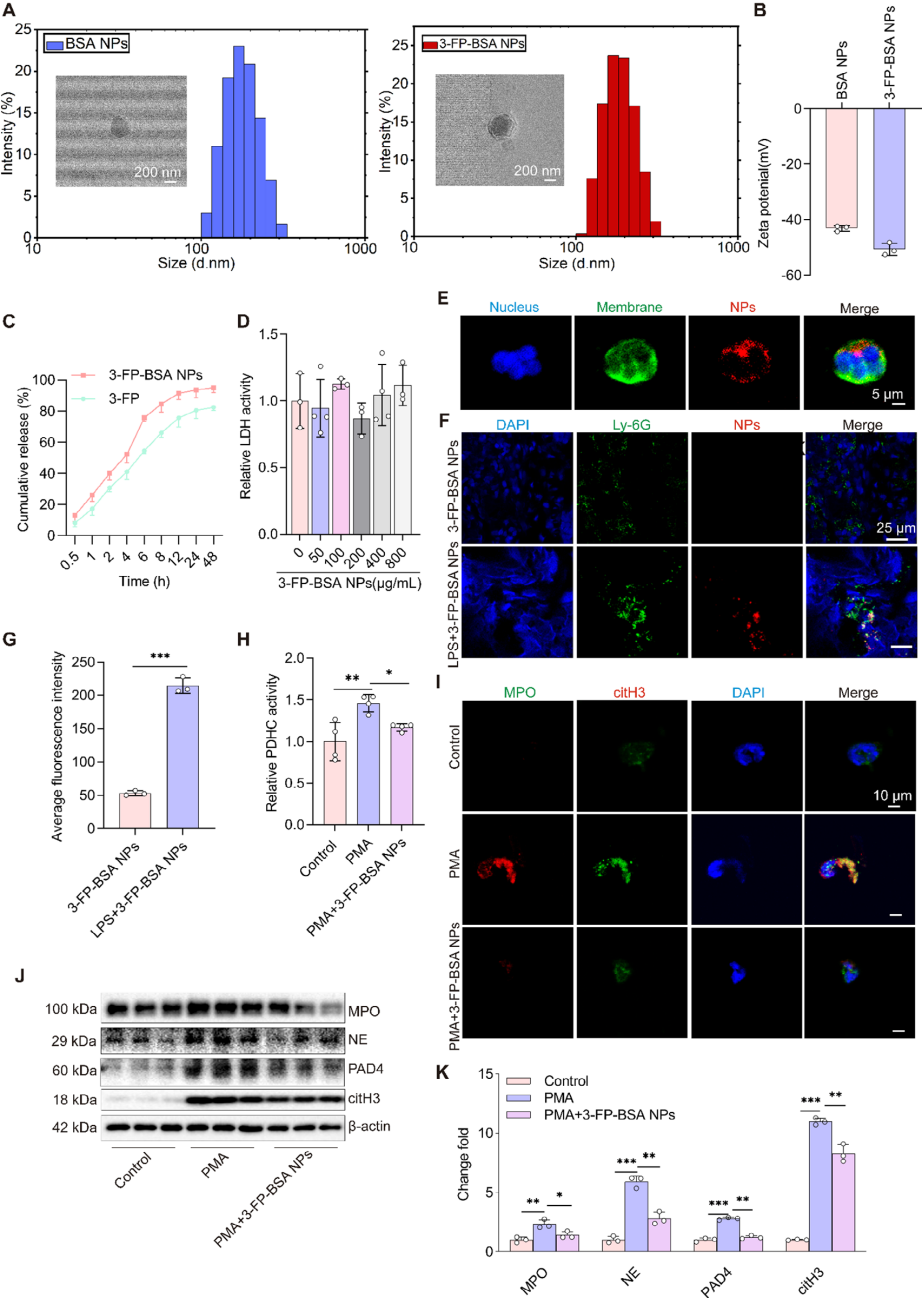
Synthesis and characterization of PDHA1‐targeted nanoparticles to inhibit NETosis. (A) Size distribution and TEM images of BSA NPs and 3‐FP‐BSA NPs. (B) The zeta potential of BSA NPs and 3‐FP‐BSA NPs. *n* = 3. (C) The release of 3‐FP solutions from 3‐FP‐BSA NPs in PBS. (D) Cytotoxicity analysis of 3‐FP‐BSA NPs to mouse bone marrow‐derived neutrophils. (E) Confocal images of Cy5‐3‐FP‐BSA NPs uptake by mouse bone marrow‐derived neutrophils in vitro, Scale bar = 5 µm. (F,G) Mice were pretreated intravenously with 3‐FP‐BSA NPs (2.5 mg/kg) and then subjected to ALI via intratracheal LPS instillation, followed by euthanasia 12 h post‐LPS challenge, *n* = 4. IF images of Cy5‐3‐FP‐BSA NPs uptake by neutrophils in vivo, scale bar = 25 µm. (H) PDHC activity was measured by a commercially available PDHC activity assay kit. *n* = 4. (I) IF for MPO (red) and citH3 (green) protein expression in treated mouse bone marrow‐derived neutrophils, scale bar = 10 µm. (J,K) Western blot for MPO, NE, PAD4, and citH3 in 3‐FP‐BSA NPs treated mouse bone marrow‐derived neutrophils after PMA stimulation, *n* = 3. Statistical significance was determined by Student's *t*‐test (two‐tailed, unpaired) or one‐way ANOVA. *p < 0.05, **p < 0.01, and ***p < 0.001.

### 3.7 3‐FP‐BSA NPs Alleviate LPS‐induced ALI in Mice

Next, the therapeutic efficacy of 3‐FP‐BSA NPs in ALI mice was evaluated. Micro‐computed tomography (Micro‐CT) analysis indicated that 3‐FP‐BSA NPs‐treated mice exhibited a significant reduction in areas of segmental consolidation following LPS challenge (Figure 7A). 3D reconstructions further corroborated the structural alterations, revealing markedly increased airspace volumes in 3‐FP‐BSA NPs‐treated mice (Figure 7A). Histological examination demonstrated a significant alleviation of lung injury in the 3‐FP‐BSA NP‐treated group, as evidenced by diminished leukocyte infiltration, alveolar congestion, and thickening of the alveolar barrier compared with LPS‐challenged ALI mice (Figure 7B,C). Moreover, treatment with 3‐FP‐BSA NPs resulted in reduced serum LDH activity (Figure 7D), indicating mitigation of pulmonary cellular damage. Serum TNF‐α levels were also significantly lower in the 3‐FP‐BSA NP‐treated group relative to the ALI mice (Figure 7E). Furthermore, mice receiving 3‐FP‐BSA NPs showed marked downregulation of key inflammatory mediators related to ALI, including mRNA expressions of *Il1b*, *Inos*, *Ccl2*, and *Il6*, whereas the anti‐inflammatory cytokine *Il10* was significantly upregulated (Figure 7F). Our previous studies have identified the NLR family pyrin domain‐containing 3 (NLRP3) inflammasome as a novel mechanism driving inflammatory amplification in ALI [14, 42, 43]. The protein expression levels of NLRP3, pro‐interleukin‐1β (IL‐1β), and IL‐1β p17 in the lungs were significantly suppressed following 3‐FP‐BSA NP treatment (Figure 7G,H). We further investigated the role of PDHA1 in NETosis formation during ALI in vivo. ELISA results indicated that LPS‐challenged mice treated with 3‐FP‐BSA NPs exhibited significantly lower levels of both MPO‐dsDNA complexes and free dsDNA in serum compared with untreated ALI mice (Figure 7I,J). Consistent with this, Western blot analysis showed a marked reduction in the expression of NETosis‐related proteins MPO, NE, PAD4, and citH3 in the 3‐FP‐BSA‐NP treatment (Figure 7K,L). These results suggest that PDHA1 in neutrophils plays a critical role in the pathophysiology of organ dysfunction associated with ALI.

**FIGURE 7:**
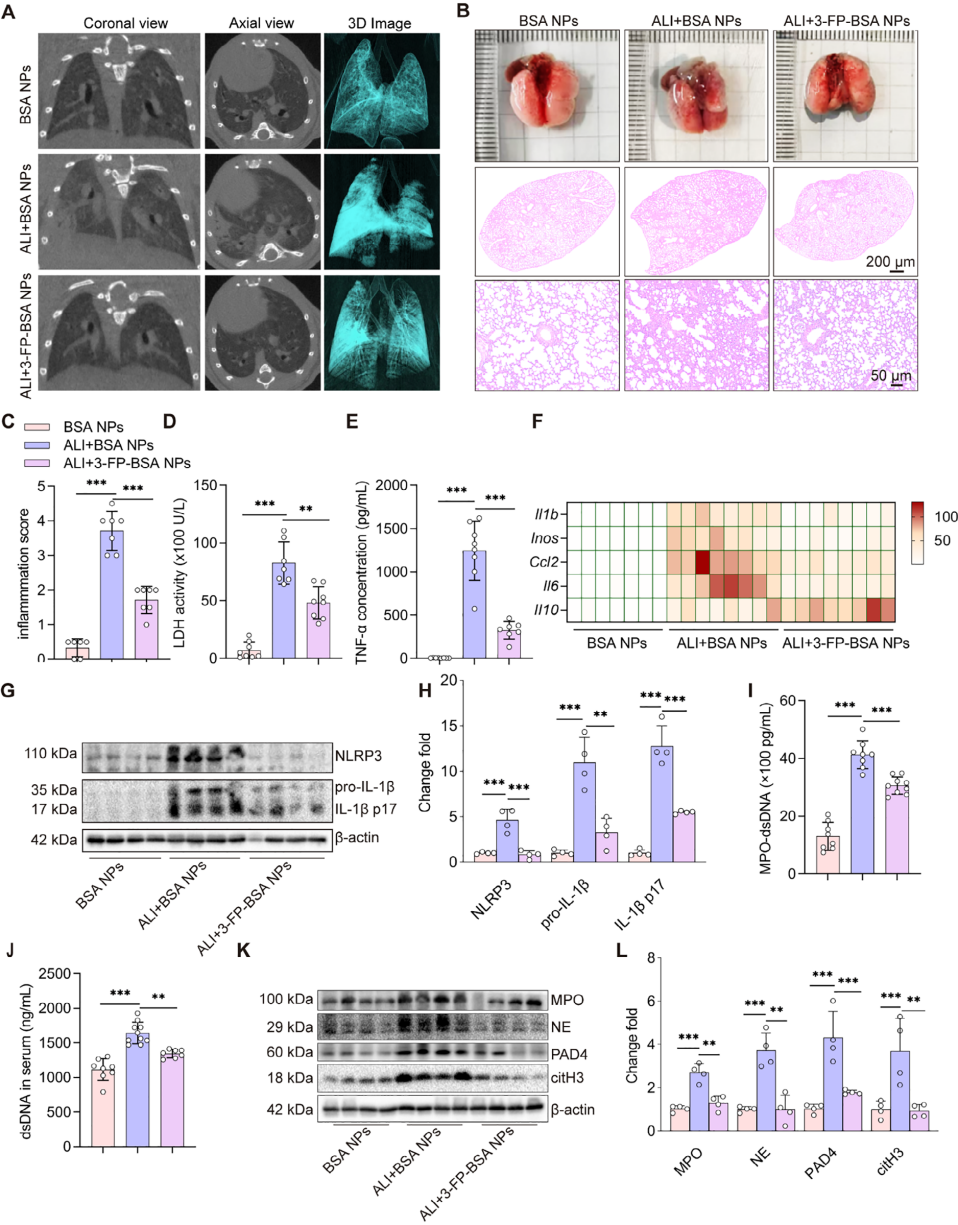
Nanoparticles that target PDHA1 alleviate LPS‐induced ALI in mice by inhibiting neutrophil NETosis. (A) High‐resolution micro‐CT was utilized to assess pulmonary structural changes in a murine model of ALI. Multiplanar reconstructions, including coronal and axial views, were obtained at the level of the T6 thoracic vertebra. Representative 3D reconstructions were generated from in vivo‐acquired images, with voxel intensities thresholded within a Hounsfield unit (HU) range of −1000 to −400, *n* = 6 mice/group. (B) Lung appearance and HE staining after treatment with 3‐FP‐BSA NPs. (C) Inflammation score was assessed, *n* = 6–7. (D) The LDH level in serum was detected, *n* = 6–8. (E) ELISA was used to detect the level of TNF‐α in serum, *n* = 6–8. (F) Heatmap showing the relative mRNA expression levels of *Il1b*, *Inos, Ccl2, Il6, and Il10* in lung tissue was determined by Real‐time PCR, *n* = 6–8. (G,H) Western blot for NLRP3, pro‐IL‐1β, and IL‐1β p17 in lung tissue. (I) The level of MPO‐dsDNA content in serum. (J) The level of dsDNA content in the serum, *n* = 6–8. (K,L) Western blot for MPO, NE, PAD4, and citH3 in lung tissue. Statistical significance was determined by one‐way ANOVA. **p < 0.01 and ***p < 0.001.

### 3.8 PDHA1 Hyperactivation Induces mtROS to Promote NETosis Through Mitochondrial Dysfunction

We next investigated the mechanism by which PDHA1 hyperactivation promoted NETosis. Given that phosphorylation of PDHA1 at the inhibitory Ser293 site is catalyzed by PDK1 [44], we used the PDK1 inhibitor AZD7545 to activate PDHA1. Treatment with AZD7545 reduced PDHA1 phosphorylation at Ser293 (Figure S7A,B), consistent with enhanced PDHC activity (Figure S7C). To identify downstream pathways, RNA‐sequencing was performed on bone marrow‐derived neutrophils undergoing AZD7545‑induced NETosis. KEGG pathway analysis revealed significant upregulation of the ROS signaling pathway (Figure 8A), which was further supported by GSEA of the transcriptomic data (Figure 8B). These findings suggest that an overactive OXPHOS system imposed a substantial oxidative burden on mitochondria, leading to excessive mtROS production. Our studies found that AZD7545‐induced NETosis was associated with a concurrent increase in mtROS levels, as demonstrated by mitoSOX staining (Figure 8C) and flow cytometric analysis (Figure 8D,E), along with a marked loss of mitochondrial membrane potential indicated by decreased JC‐1 aggregate/monomer fluorescence (Figure 8F). Inhibition of PDHA1 with 3‑FP before PMA stimulation preserved JC‑1 staining (Figure S8A) and attenuated mtROS production (Figure S8B–D). Moreover, the mtROS scavenger MitoQ substantially suppressed the upregulation of NETosis‐associated proteins induced by the PDHA1 activating AZD7545, including MPO, NE, PAD4, and citH3 (Figure 8G–I). MitoQ also decreased levels of dsDNA and MPO‐dsDNA complexes in the supernatants after PDHA1 activation (Figure 8J,K). Collectively, PDHA1 hyperactivation disrupts mitochondrial homeostasis, producing excessive mtROS, which results in NETosis in neutrophils.

**FIGURE 8:**
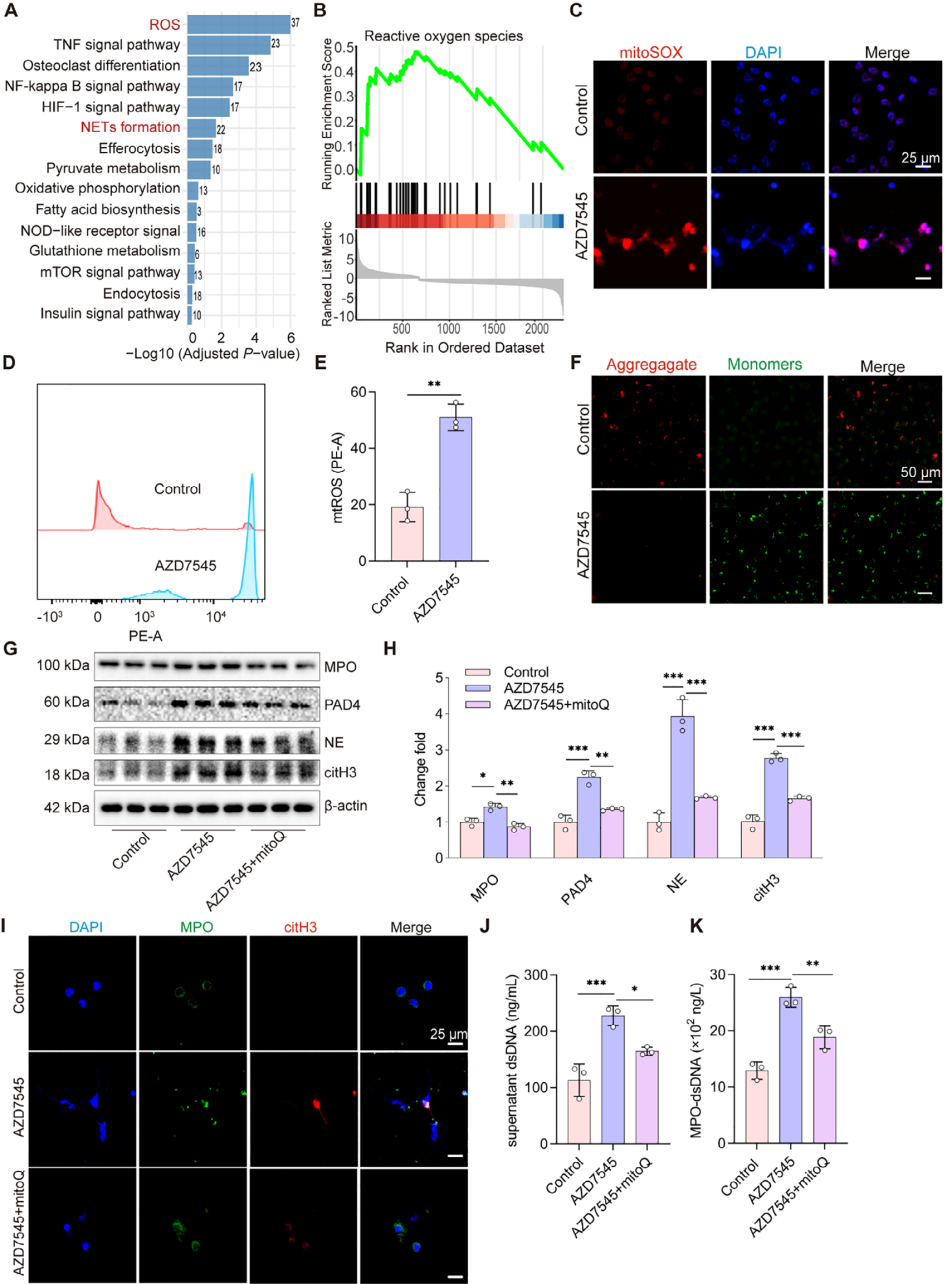
PDHA1 hyperactivation induces mtROS to promote NETosis via mitochondrial dysfunction. Mouse bone marrow‐derived neutrophils were treated with PDHA1 activator AZD7545 (10 µM) for 4 h. RNA‐sequencing analysis of NETosis induced by PDHA1 hyperactivation was performed. (A) KEGG pathway enrichment analysis indicated an upregulation of the ROS signaling pathway in AZD7545‐treated mouse bone marrow‐derived neutrophils. (B) GSEA analysis shows that the ROS signaling pathway was activated in AZD7545‐treated mouse bone marrow‐derived neutrophils. (C) Representative IF images of mtROS in mouse bone marrow‐derived neutrophils detected by mitoSOX (red) and DAPI (blue); scale bar = 25 µm. (D,E) Flow cytometric analysis (D) and quantification (E) of mtROS levels in neutrophils. (F) Representative images of JC‐1 staining showing mitochondrial membrane potential. Red fluorescence (aggregates) and green fluorescence (monomers) indicate intact and depolarized mitochondrial membrane potential; scale bar = 50 µm. (G,H) Western blot for MPO, NE, PAD4, and citH3 in mouse bone marrow‐derived neutrophils treated with mitoQ before AZD7545 administration. (I) IF for MPO and citH3 protein expression in treated neutrophils, scale bar = 25 µm. (J) The level of dsDNA content in the supernatant. (K) The level of MPO‐dsDNA content in the supernatant, *n* = 3. Statistical significance was determined by Student's *t*‐test (two‐tailed, unpaired) or one‐way ANOVA. *p < 0.05, **p < 0.01, and ***p < 0.001.

### 3.9 AMPK‑Mediated PDHA1 Activation Promotes NETosis

We next sought to elucidate the mechanism driving PDHA1 hyperactivation during neutrophil NETosis. To systematically identify upstream regulators of PDHA1, we performed transcriptomic sequencing on PMA‐stimulated mouse bone marrow‐derived neutrophils. KEGG pathway analysis highlighted significant enrichment of the AMPK signaling pathway (Figure 9A). Subsequent phosphoproteomic profiling of human peripheral blood neutrophils confirmed AMPK activation, as indicated by enhanced phosphorylation at its key activating residue, T183 (Figure 9B). Given the homology between human AMPK T183 and murine T172 [45], we examined the conserved T172 site by Western blot and confirmed its elevated phosphorylation in peripheral blood neutrophils from ALI mice (Figure 9C,D). A recent study demonstrated that AMPK directly phosphorylates PDHA1 at Ser314, thereby disrupting PDHA1‐PDK1 interaction and preventing phosphorylation of PDHA1 Ser293 [36]. Accordingly, we constructed a PDHA1 S314A mutant plasmid so that AMPK cannot phosphorylate at this residue. dHL‐60 cells were transfected with either PDHA1‑WT or the PDHA1‑S314A mutant. Co‑IP revealed that the PDHA1‑S314A mutation enhanced the interaction between p‑PDHA1 Ser293 and PDK1 in PMA‑induced dHL‑60 cells (Figure 9E). In PMA‐stimulated neutrophils expressing the PDHA1‑S314A mutant, PDHA1 Ser293 phosphorylation remained elevated compared with PMA‐treated PDHA1‑WT Controls; PDHC activity was significantly reduced, and NETosis formation was markedly attenuated (Figure 9F–H). Moreover, AMPK inhibition with Compound C also increased the interaction between p‑PDHA1 Ser293 and PDK1 (Figure S9), indicating that AMPK negatively regulates the PDHA1‐PDK1 interaction. Inhibition of AMPK in PMA‑induced mouse bone marrow‐derived neutrophils markedly increased PDHA1 Ser293 phosphorylation (Figure 9I,J). Concomitantly, AMPK inhibition reduced the levels of NETosis‐associated proteins (Figure 9I–K) and extracellular levels of dsDNA and MPO‐dsDNA complexes (Figure 9L,M). Together, these findings confirm that AMPK activation promotes PDHA1 Ser314 phosphorylation, which disrupts PDHA1‑PDHK1 binding, reduces PDHA1 Ser293 phosphorylation, and ultimately drives selective metabolic reprogramming toward OXPHOS and NETosis.

**FIGURE 9:**
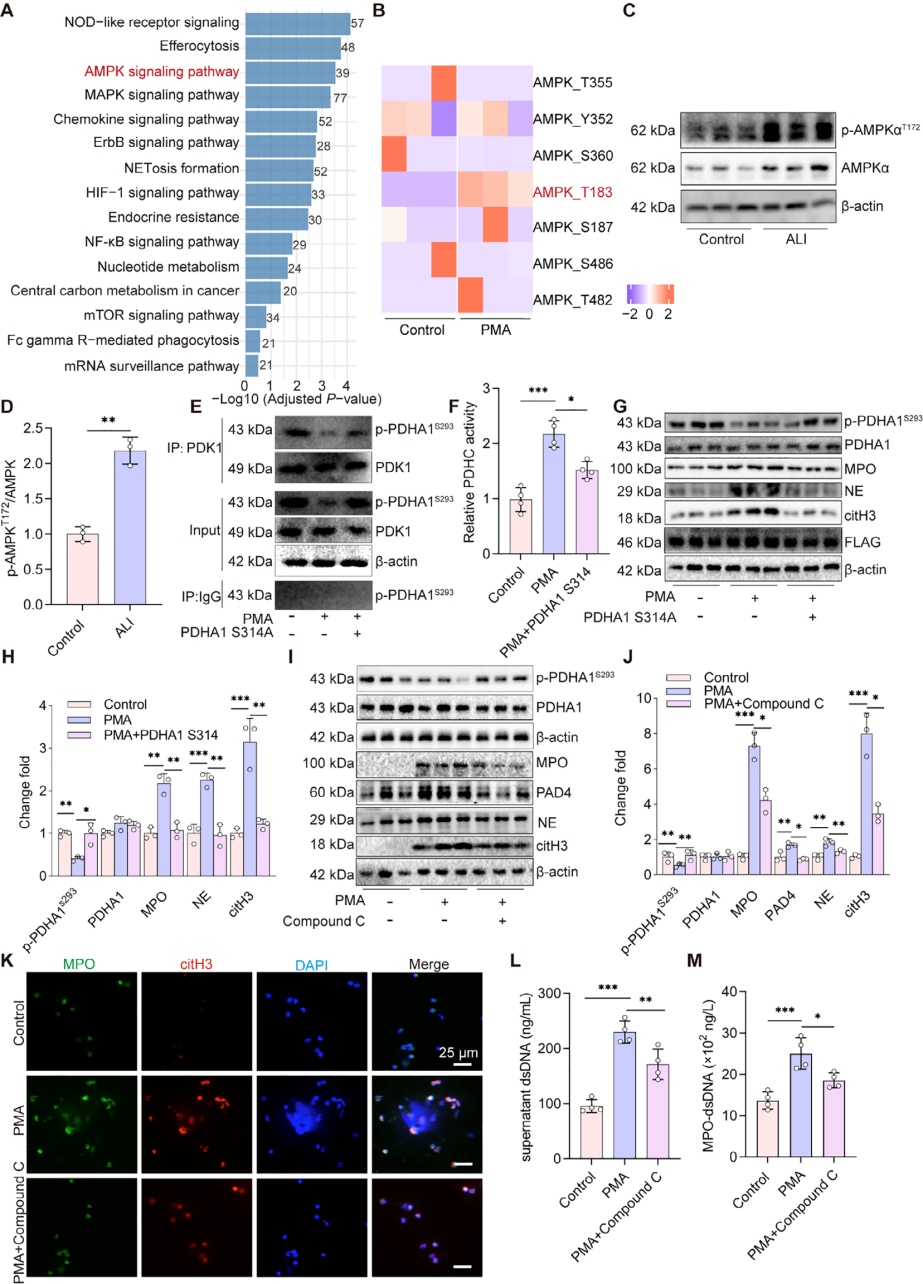
AMPK‑mediated PDHA1 activation promotes NETosis. (A) KEGG pathway enrichment analysis of transcriptomic data from PMA‑stimulated mouse bone marrow‐derived neutrophils highlighted significant activation of the AMPK signaling pathway. (B) Phosphoproteomic profiling confirms AMPK activation in human peripheral blood neutrophils, as shown by increased phosphorylation at its key activating site T183. (C,D) Western blot analysis validates elevated phosphorylation of the conserved murine AMPK site T172 in peripheral blood neutrophils isolated from LPS‐induced ALI mice. (E) In PMA‑stimulated dHL‑60 cells expressing the PDHA1‑S314A mutant, Co‐IP analysis of the interaction between PDK1 and p‐PDHA1^S293^. (F) PDHC activity was significantly reduced in PMA‑stimulated dHL‑60 cells expressing the PDHA1‑S314A mutant. G‐H) Elevated p‐PDHA1^Ser293^ and attenuated NETosis in PMA‐treated PDHA1‐S314A mutant cells relative to PDHA1‐WT controls. (I,J) AMPK inhibition (Compound C) enhanced PDHA1 Ser293 phosphorylation and attenuated the expression of NETosis‑associated proteins in PMA‑induced mouse bone marrow‐derived neutrophils. (K) IF for MPO and citH3 protein expression in Compound C‐treated mouse bone marrow‐derived neutrophils, scale bar = 25 µm. L‐M) Extracellular levels of dsDNA and MPO‑dsDNA complexes are reduced upon AMPK inhibition, *n* = 3–4. Statistical significance was determined by Student's *t*‐test (two‐tailed, unpaired) or one‐way ANOVA. *p < 0.05, **p < 0.01, and ***p < 0.001.

## 4 Discussion

The aberrant activation of PDHA1, mediated by its dephosphorylation at Ser293, emerged as a central driver of pathogenic NETosis. We found that this activation enforced a selective metabolic shift characterized by enhanced OXPHOS and limited glycolysis in neutrophils from ALI/ARDS. The resulting mitochondrial dysfunction, marked by impaired homeostasis and elevated mtROS, directly fueled NETosis formation. Critically, this pathway was governed by AMPK, which directly phosphorylates PDHA1 at Ser314, disrupting the PDHA1‐PDK1 interaction and thereby preventing PDK1 from phosphorylating PDHA1 Ser293. Importantly, therapeutic inhibition of PDHA1 via a neutrophil‐targeted nanoparticle alleviated LPS‐induced ALI. In summary, this work delineates a pivotal metabolic axis controlling neutrophil NETosis and presents a PDHA1‐targeted intervention strategy with therapeutic potential for ALI/ARDS (Figure 10).

**FIGURE 10:**
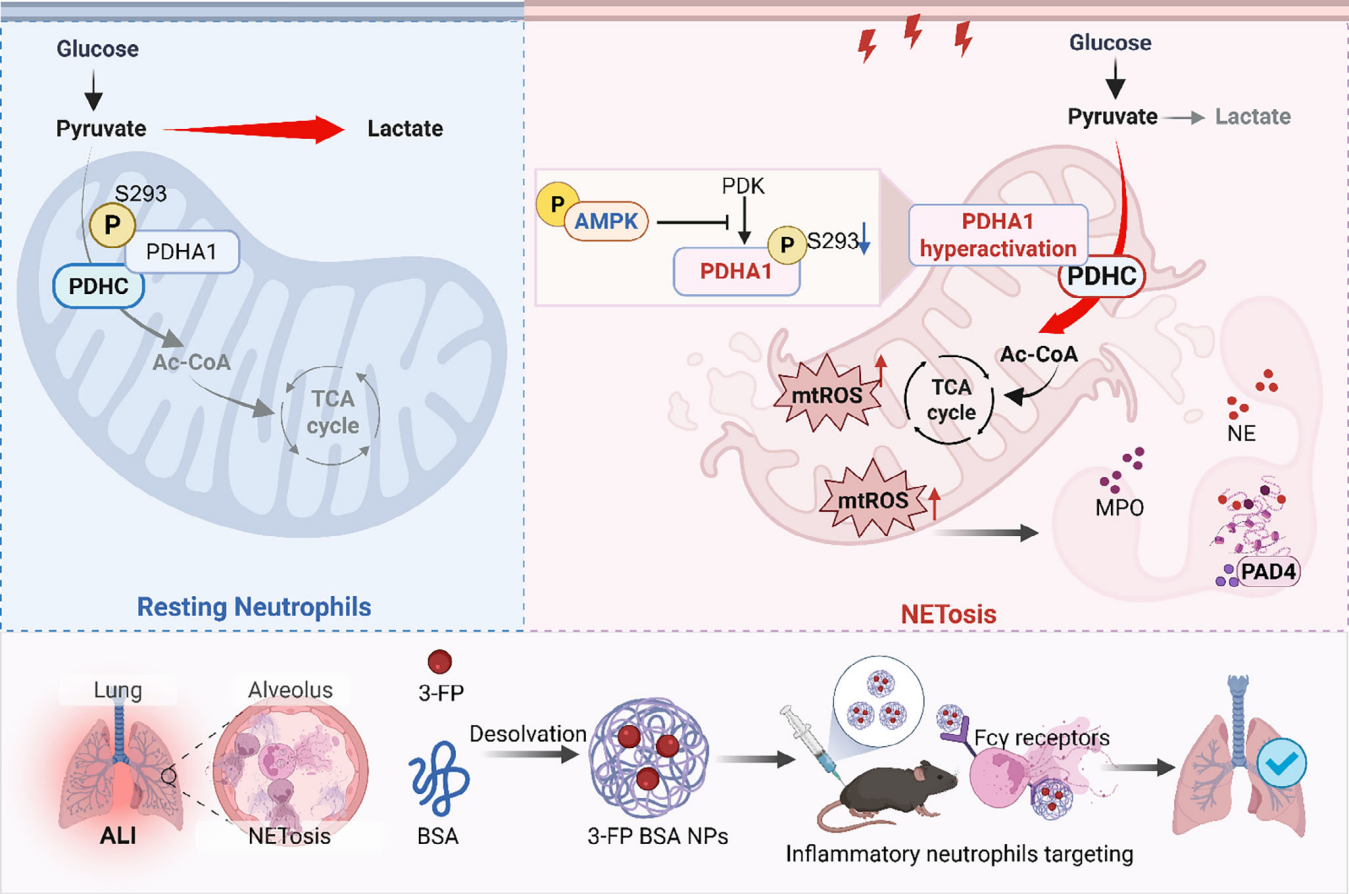
Schematic illustration. During NETosis, neutrophils shift metabolically toward enhanced OXPHOS and partially reduced glycolysis. This shift is driven by PDHA1 hyperactivity‐dependent selective metabolic reprogramming. The neutrophil‐targeted nanoparticle 3‑FP‑BSA NPs effectively inhibit NETosis and attenuate ALI.

This study identifies PDHA1 hyperactivation‐mediated glucose metabolism reprogramming as a previously unrecognized mechanistic driver of neutrophil NETosis. PMA is a standard inducer of nicotinamide adenine dinucleotide phosphate (NADPH) oxidase‑dependent suicidal NETosis, closely recapitulating the NETosis observed in LPS‑induced ALI [46, 47]. Although PMA is a non‑physiological stimulus, the key selective metabolic shift has been validated in neutrophils from ALI mice. Neutrophil NETosis requires considerable cytoskeletal reorganization and metabolic energy. Although neutrophils primarily rely on glycolysis and display low TCA cycle and OXPHOS activity, metabolic flexibility may underlie neutrophil heterogeneity [16]. Advances in metabolomic technologies are expanding our insight into neutrophil immunometabolism. Altered neutrophil metabolic states have been reported in inflammatory diseases such as diabetes [48], cystic fibrosis [49], and atherosclerosis [50]. In this study, we systematically profiled the neutrophil metabolome to elucidate the metabolic basis of neutrophil function in ALI. We found that neutrophils from both ARDS patients and ALI mice consistently exhibited upregulated oxidative glucose metabolism coupled with downregulated glycolysis. We propose that the activation of mitochondrial metabolism primarily serves the burst‑phase demands of NETosis, including ROS generation and the provision of energy and biosynthetic precursors, rather than simply replacing glycolysis for ATP production. Notably, the enhanced respiratory activity observed during NETosis appeared to be driven by increased PDHA1 function, as indicated by a specific reduction in phosphorylation at Ser293. This dephosphorylation promotes the entry of pyruvate into the mitochondrial TCA cycle via acetyl‑CoA. Among the known regulatory phosphorylation sites of PDHA1, Ser293 is recognized as the dominant regulator of PDH complex activity due to its rapid kinetics and potent inhibitory effect, in contrast to the slower and weaker modulation mediated by Ser300/232 phosphorylation [35, 51, 52]. Our data thus link NETosis to a specific PDHA1‐mediated shift toward mitochondrial oxidative metabolism. The role of PDHA1 extends beyond immune regulation. Recent work in cancer biology has shown that impaired PDHC activity resulting from PDHA1 deficiency does not affect primary tumor growth but severely suppresses metastasis, identifying PDHA1 as a previously unrecognized driver of tumor progression, at least in breast cancer models [36]. PDHA1 activation modulates energy metabolism to attenuate cardiac ischemia and reperfusion injury [53]. Our recent study demonstrated that PDHA1 hyperactivation in pulmonary endothelial cells promotes endothelial senescence through metabolic reprogramming [54]. This highlights the context‐dependent functions of PDHA1 in coordinating metabolic flux to support demanding cellular processes.

Leveraging the well‑documented ability of BSA NPs to enhance drug biocompatibility and reduce systemic toxicity [55], we encapsulated the PDHA1 inhibitor 3‑FP into BSA NPs for targeted mitigation of NETosis in ALI. This approach is further supported by BSA's capacity to promote transmembrane drug delivery by binding to Fcγ receptors, which are highly expressed on activated neutrophils [56]. Uptake of these denatured BSA NPs occurs through an activation‑dependent mechanism: only neutrophils that have adhered to inflamed endothelium, not resting circulating cells, efficiently internalize the particles, primarily via FcγRIII recognition of the denatured albumin as an immune‑complex‑like structure [38, 57]. Once loaded, neutrophils can migrate across the vascular endothelium into inflamed tissue, thereby acting as active drug carriers that deliver 3‑FP specifically to sites of NETosis. This strategy enables precise therapeutic targeting while sparing non‑inflamed tissue and preserving the physiological functions of circulating neutrophils. Our findings indicate that heightened PDHA1 activity in lung tissue from ARDS patients drives NETosis and contributes to disease progression. The administration of 3‑FP‑BSA NPs effectively suppressed NETosis and alleviated ALI in experimental models. Mechanistically, PDHA1 inhibition redirects pyruvate flux away from oxidative metabolism. PDHA1 inhibition likely concurrently elevates the cytosolic NAD^+^/NADH ratio, resulting from heightened NAD^+^ consumption by the PDH reaction coupled with insufficient NAD^+^ regeneration [58]. Under conditions of PDHA1 hyperactivation, substantial NAD^+^ is consumed by the PDH reaction without adequate regeneration, leading to a lowered NAD^+^/NADH ratio, elevated mtROS, and subsequent mitochondrial dysfunction [59, 60]. By counteracting this hyperactive state, 3‑FP‑BSA NPs restore redox balance and reduce mitochondrial stress, thereby attenuating neutrophil‑driven tissue injury in ALI.

Our findings elucidate a mechanistic pathway in which AMPK activation, triggered during neutrophil NETosis, directly phosphorylates PDHA1 at Ser314. This modification disrupts the PDHA1‑PDK1 interaction, thereby preventing PDK1 from accessing and phosphorylating the inhibitory Ser293 site on PDHA1 [36]. Consequently, PDHA1 becomes hyperactive, driving a non‑canonical selective metabolic reprogramming characterized by enhanced oxidative phosphorylation, which ultimately fuels NETosis. This regulatory axis establishes a critical link between energy‐sensing signaling and pyruvate metabolism. AMPK is broadly recognized for its role in directing cell fate decisions, such as modulating apoptosis, autophagy, endoplasmic reticulum stress, and ROS generation [61]. Previous studies indicate that AMPK localizes to the mitochondrial matrix, where it phosphorylates the catalytic alpha subunit of PDHA at two key residues, S295 and S314. This phosphorylation activates PDH enzymatic activity by alleviating its inhibitory phosphorylation by PDHKs [36]. Our data highlight a specific function of AMPK in fine‑tuning PDHA1 activity via Ser314 phosphorylation and PDK1 disengagement in neutrophils. In cardiac protection, for instance, AMPK signaling contributes to the beneficial effects of dichloroacetate (DCA) against myocardial infarction, partly through PDH activation [62]. In contrast, we observed that during ALI, the AMPK‑PDHA1 axis drives a pro‑inflammatory metabolic shift that promotes NETosis. Interestingly, the consequences of modulating this pathway appear context‐dependent. In tumor settings, inhibition of PDK1 activity, which likewise reduces PDHA1 Ser293 phosphorylation and enhances TCA cycle flux, has been reported to promote cancer cell survival [63]. This contrast underscores the cell‐type and disease‐specific outcomes of metabolic rewiring. Further supporting a role in cell survival, AMPK activation has been shown to inhibit chromium‑induced neutrophil apoptosis [64], whereas Ampkα knockout promotes hepatocyte apoptosis [65]. Collectively, these observations position the AMPK‑PDHA1 axis as a versatile regulator of metabolism and function across different physiological and pathological states.

Several limitations should be considered in this study. First, we acknowledge that NADPH oxidase‑independent pathways exist, such as ionomycin‑induced and vital NETosis pathways [66]. Our study focuses on the NADPH oxidase‑dependent pathway; whether PDHA1 hyperactivation‑mediated glucose‐selective metabolic reprogramming fuels NETosis in other pathways remains unknown. Second, the mechanism by which PDHA1 hyperactivation promotes NETosis is unclear. Our metabolomics data showed succinate and citrate accumulation in NETotic neutrophils, which in macrophages causes mitochondrial dysfunction and ROS production [67]. However, whether this occurs in neutrophils and the precise source of mitochondrial ROS (complex I vs. III, possibly via reverse electron transport) requires further investigation [68]. Third, terminally differentiated primary neutrophils are notoriously refractory to genetic manipulation, necessitating the use of dHL‐60 cells for plasmid‐based site‐directed mutagenesis (S293A and S314A) and gene knockdown experiments. Future work will leverage neutrophil‐targeted conditional knock‐in mice (*Pdha1* ^S293A/fl^ crossed with *S100A8*‐Cre) to evaluate whether endogenous PDHA1 hyperactivation exacerbates neutrophil NETosis in LPS‐induced ALI. Larger independent cohorts are needed to confirm clinical relevance and correlate PDHA1 phosphorylation with disease severity.

## 5 Conclusion

Collectively, our study demonstrated that PDHA1 hyperactivation‐dependent selective metabolic shift is a critical driver of NETosis in neutrophils from ALI mice and ARDS patients. We developed a targeted nanoplatform to specifically reduce NETosis‐neutrophil accumulation, offering a promising therapeutic strategy for ALI. Mechanistically, PDHA1 hyperactivation disrupts mitochondrial homeostasis, and an elevation of mitochondrial membrane potential contributes to driving mtROS production. This study thus unveils a new regulatory mechanism in NETosis, proposing the targeting of PDHA1 hyperactivation as a viable strategy against ALI.

## Author Contributions

Y.B.L., W.J.Z., C.Y.Z., M.R.C., N.S.Y.Y., H.X.S., Y.L.Q., H.C., J.T.Y., L.Y.L., and J.X.D. performed the experiments. Y.B.L., W.J.Z., N.S.Y.Y., J.T.Y., and C.Y.Z. analyzed the data. J.X.D., J.B.X., S.K.L., C.X.G., and Y.Z. contributed reagents/materials/analysis tools. W.J.Z. and Y.B.L. wrote the paper. Y.Z. and J.X.D. conceived, designed the experiments, and critically reviewed the manuscript. All authors had final approval of the submitted versions.

## Funding

This work was supported by the National Natural Science Foundation of China (82370086, 82301756, 82400108, and 82402551), Postdoctoral Fellowship Program of CPSF (GZC20233159), the Natural Science Foundation of Hunan province, China (2025JJ60690), the Open competition mechanism to select the best candidates for key research projects of Ningxia Medical University (XJKF230104 and XJKF240306), and the Independent Exploration and Innovation Project for Postgraduate Students in Hunan Province (CX20230274).

## Ethics Statement

All animal procedures performed in this study were conducted in accordance with the regulatory standards established by the Committee on the Use and Care of Animals of Central South University (2023‐KT026). Scientific and ethical approval for human data and the statement are described below. The institutional review board at Xiangya Hospital approved this study, with a waiver of informed consent (No.2022100983).

## Conflicts of Interest

The authors declare no conflicts of interest.

## Supporting information




**Supporting File**: advs78307‐sup‐0001‐SuppMat.docx.

## Data Availability

The datasets supporting the conclusions of this article are included within the article and its additional file. For any further data requests, please get in touch with the corresponding author.
