# Supplementary material for: PDHA1 Hyperactivation‐Mediated Glucose Metabolism Reprogramming Fuels Neutrophil NETosis and Promotes Acute Lung Injury
Source: Adv Sci (Weinh). 2026 Oct 14:e78307. Online ahead of print. doi: 10.1002/advs.78307 (PMC13669207; doi:10.1002/advs.78307)
Supplement: Supplementary file 1 [file ADVS-9999-e78307-s001.docx]

**Supporting Information**

**Supplementary Figures**

**Figure S1. Integrated analysis of marker gene expression identifies neutrophils in ARDS patients (GSE157789).**

A) Dot plots showing the percentage of cells expressing specific marker genes and the average expression level across major immune cell types, including B cells, T cells, NK cells, Monocytes, Neutrophils, and DCs. B) Dot plot of LDN-associated markers (CD35, CD11b, CD66b, and CD16) expression across major immune cell types. C) Violin plots displaying the expression level distributions of CSF3R, FCGR3B, and CXCR2 across annotated cell types. D) UMAP plots showing the spatial distribution and expression heatmap of CSF3R, FCGR3B, and CXCR2 within the integrated single-cell landscape.

**Figure S2. Enhanced OXPHOS in neutrophils from ARDS patients.**

A) UMAP projection illustrating the clustering of 10 distinct cell populations in lung tissue samples from ARDS patients (GSE223793). B) KEGG pathway enrichment analysis was performed on the neutrophils. C-D) Western blot was used to detect p-PDHA1 S293, MPO, NE, and citH3 expression in peripheral blood neutrophils isolated from ARDS patients. E-F) OXPHOS-related proteins in peripheral blood neutrophils from ARDS patients and HCs were detected by Western blot. G) Analysis of lactate production in peripheral blood neutrophils from ARDS patients, *n* = 6. Statistical significance was determined by Student's t-test (two-tailed, unpaired). **P* < 0.05, ***P* < 0.01, and ****P* < 0.001.

**Figure S3. PCA and volcano plot analyses of PMA‑treated human peripheral blood neutrophils.**

A) PCA score plot of all samples. B) Volcano plot of significantly changed features between the PMA and Control groups.

**Figure S4. Purity assessment of isolated neutrophils from human peripheral blood, mouse peripheral blood, and mouse bone marrow.**

A) Human peripheral blood neutrophils (CD16⁺/CD11b⁺). B) Viability of human peripheral blood neutrophils assessed by 7-AAD staining. C) Mouse peripheral blood neutrophils (Ly6G⁺/CD11b⁺). D) Viability of mouse bone marrow neutrophils assessed by 7-AAD staining. E) Mouse bone marrow neutrophils (Ly6G⁺/CD11b⁺). F) Summary of purity percentages for mouse bone marrow neutrophils.

**Figure S5. Silencing PDHA1 inhibits neutrophil NETosis in dHL-60 cells.**

dHL-60 cells were treated with Ctrl shRNA or Pdha1 shRNA for 4-6 h, followed by 48 h incubation in a complete medium, and then exposed to PMA. Cell samples were harvested after 4 h. A-B) Western blot for PDHA1, MPO, NE, PAD4, and citH3 in *Pdha1* shRNA-treated dHL-60 cells, *n* = 3. C) IF for MPO and citH3 protein expression in *Pdha1* shRNA-treated dHL-60 cells, scale bar = 25 μm. D) The level of MPO-dsDNA content in dHL-60 cells treated with *Pdha1* shRNA, *n* = 3. E) The integrity of neutrophil cell membranes was detected by FM4-64; scale bar = 10 μm. F) The level of dsDNA content in the supernatant, *n* = 3. Statistical significance was determined by one-way ANOVA. **P* < 0.05, ***P* < 0.01, and ****P* < 0.001.

**Figure S6. Targeted PDHA1 nanoparticles inhibit neutrophil NETosis.**

A) Schematic diagram of BSA nanoparticle preparation. B-C) Biocompatibility of BSA NPs and 3-FP-BSA NPs. D) IF images of Cy5-3-FP-BSA NPs uptake by macrophages in the lung tissue of ALI mice. E) The level of dsDNA content in the supernatant of 3-FP-BSA NPs treated mouse bone marrow‑derived neutrophils after PMA stimulation, *n* = 3. Statistical significance was determined by one-way ANOVA. ***P* < 0.01 and ****P* < 0.001.

**Figure S7. AZD7545 significantly suppresses p-PDHA^S293^.**

Mouse bone marrow‑derived neutrophils were treated with PDHA1 activator AZD7545 (10 μM) for 4 h. A) The level of p-PDHA^S293^ was detected by Western blot. β-actin was used as the loading control. B) Quantification of the indicated protein levels in A. C) PDHC activity was measured by a commercially available PDHC activity assay kit, *n* = 3. Student's *t*-test (two-tailed, unpaired) was used to compare the Control and AZD7545. ***P* < 0.01 and ****P* < 0.001.

**Figure S8. Inhibition of PDHA1 suppresses mtROS in PMA-treated neutrophils.**

Mouse bone marrow‑derived neutrophils were exposed to the PDHA1 competitive inhibitor 3-FP before PMA administration. A) Representative images of JC-1 staining showing mitochondrial membrane potential in Mouse bone marrow‑derived neutrophils. A decrease in the red/green fluorescence ratio indicates mitochondrial depolarization; scale bar = 50 μm. B) Representative images of mtROS detection; scale bar = 25 μm. C-D) Detection of mtROS content and statistical chart by flow cytometry, *n* = 3. Statistical significance was determined by one-way ANOVA. **P* < 0.05 and ****P* < 0.001.

**Figure S9.** Compound C enhances the PMA-induced interaction between PDK1 and p-PDHA1^S293^.

Co-IP analysis shows that Compound C enhanced the PMA-induced interaction between PDK1 and p-PDHA1 Ser293. IgG was used as a negative control.

**Supplementary Tables**

Table S1. Antibody sources and dilutions.

| **Antibodies** | **Source** | **Catalog** | **Dilution** |
| --- | --- | --- | --- |
| **Primary antibodies for Western blot or IF/IP** |  |  |  |
| Anti-β-Actin monoclonal antibody | Servicebio | GB15003 | 1:2000 |
| Mouse anti-MPO antibody | Proteintech | 66177-1-Ig | 1:2000 |
| Mouse-anti-PADI4 (A-11) antibody | SANTA CRUZ | sc-365369 | 1:1000 |
| Anti-Histone H3 (citruline R2+R8+R17) | Abcam | ab5013 | 1:2000 |
| Anti-ELANE antibody | Abclonal | A8953 | 1:1000 |
| Anti-p-PDHA1^Ser293^ polyclonal antibody | ThermoFisher | MA535866 | 1:2000 |
| Anti-p-PDHA1^Ser300^ polyclonal antibody | Proteintech | 29583-1-AP | 1:2000 |
| Anti-p-PDHA1^Ser232^ polyclonal antibody | Proteintech | 81491-1-RR | 1:2000 |
| Anti-PDHA1 polyclonal antibody | ThermoFisher | 9H9AF5 | 1:2000 |
| Anti-p-AMPKα^Thr172^ polyclonal antibody | CST | 50081S | 1:2000 |
| Anti-AMPKα monoclonal antibody | Proteintech | 10929-2-AP | 1:2000 |
| Anti-LDHA monoclonal antibody | Abcam | Ab52488 | 1:10000 |
| Anti-total OXPHOS complexes antibody | Abcam | ab110413 | 1:2000 |
| Anti-NLRP3 monoclonal antibody | CST | #15101 | 1:1000 |
| Anti-IL-1β polyclonal antibody | R&D | AF-401-NA | 1:2000 |
| Anti-PDK1 polyclonal antibody | Proteintech | 18262-1-AP | 1:50 |
| **Secondary antibody** |  |  |  |
| FITC goat anti-mouse IgG (H+L) | Abclonal | AS001 | 1: 400 |
| Rhodamine (TRITC) goat anti-rabbit IgG (H+L) | Abclonal | AS040 | 1: 400 |
| FITC goat anti-rabbit IgG (H+L) | Abclonal | AS011 | 1: 400 |
| HRP-conjugated goat anti-rabbit IgG | SAB | #L3012-2 | 1:5000 |
| Goat anti-Mouse IgG | SAB | L3032 | 1:5000 |
| Rabbit anti-Goat IgG | SAB | L3042-2 | 1:5000 |
| **Antibodies for Flow Cytometry** |  |  |  |
| APC anti-mouse/human CD11b Antibody | BioLegend | 101211 | 1:100 |
| PE/Cyanine7 anti-human CD11b Antibody | BioLegend | 301321 | 1:100 |
| PE/Cyanine7 anti-mouse Ly-6G/Ly-6C (Gr-1) Antibody | BioLegend | 108415 | 1:100 |
| FITC anti-human CD16 Antibody | BioLegend | 302005 | 1:100 |

Table S2. Demographic and clinical characteristics of the ARDS patients

| Demographics | ARDS (*n* = 12) |
| --- | --- |
| Age, years | 71.42 (8.89) |
| Male, *n* (%) | 8 (66.7) |
| BMI, kg/m^2^ | 23.07 (3.57) |
| PaO_2_/FiO_2_ | 127.92 (48.54) |
| SOFA score | 9.33 (4.96) |
| APACHE II score | 22.16 (10.17) |
| WBC, 10^9^/L | 14.76 (4.36) |
| NEUT, 10^9^/L | 13.2 (4.39) |
| CRP, mg/L | 125.43 (61.53) |
| PCT, ng/mL | 2.16 (1.87) |

Table S3. Sequences of the primers used to quantify gene expression

| **Primers** | **Sequence** | **Source** |
| --- | --- | --- |
| *m-Actb* | F: 5’- CACCATGTACCCAGGCATTG -3’  R: 5’- CCTGCTTGCTGATCCACATC -3’ | Sangon Biotech |
| *m-Il10* | F: 5’- GTTGCCAAGCCTTATCGGAA -3’  R: 5’- GCTCCACTGCCTTGCTCTTAT -3’ | Sangon Biotech |
| *m-Inos* | F: 5’- AACAGGGAGAAAGCGCAAAA -3’  R: 5’- CCTCACATACTGTGGACGGG -3’ | Sangon Biotech |
| *m-Il1b* | F: 5’- CAGGCAGGCAGTATCACTCA -3’  R: 5’- AGCTCATATGGGTCCGACAG -3’ | Sangon Biotech |
| *m-Ccl2* | F: 5’- GTCCCTGTCATGCTTCTGG -3’  R: 5’- CACTGGTGCTGCTGCTACTAAGA -3’ | Sangon Biotech |
| *m-Il6* | F: 5’- GTACTCCAGAAGACCAGAGG -3’  R: 5’- TGCTGGTGACAACCACGGCC -3’ | Sangon Biotech |
